# Structural analysis of Cytochrome P450 BM3 mutant M11 in complex with dithiothreitol

**DOI:** 10.1371/journal.pone.0217292

**Published:** 2019-05-24

**Authors:** Karla Frydenvang, Marlies C. A. Verkade-Vreeker, Floor Dohmen, Jan N. M. Commandeur, Maria Rafiq, Osman Mirza, Flemming Steen Jørgensen, Daan P. Geerke

**Affiliations:** 1 Department of Drug Design and Pharmacology, University of Copenhagen, Copenhagen, Denmark; 2 AIMMS Division of Molecular Toxicology, Department of Chemistry and Pharmaceutical Sciences, Vrije Universiteit, Amsterdam, the Netherlands; Universidade Nova de Lisboa Instituto de Tecnologia Quimica e Biologica, PORTUGAL

## Abstract

The bacterial Cytochrome P450 (CYP) BM3 (CYP102A1) is one of the most active CYP isoforms. BM3 mutants can serve as a model for human drug-metabolizing CYPs and/or as biocatalyst for selective formation of drug metabolites. Hence, molecular and computational biologists have in the last two decades shown strong interest in the discovery and design of novel BM3 variants with optimized activity and selectivity for substrate conversion. This led e.g. to the discovery of mutant M11 that is able to metabolize a variety of drugs and drug-like compounds with relatively high activity. In order to further improve our understanding of CYP binding and reactions, we performed a co-crystallization study of mutant M11 and report here the three-dimensional structure M11 in complex with dithiothreitol (DTT) at a resolution of 2.16 Å. The structure shows that DTT can coordinate to the Fe atom in the heme group. UV/Vis spectroscopy and molecular dynamics simulation studies underline this finding and as first structure of the CYP BM3 mutant M11 in complex with a ligand, it offers a basis for structure-based design of novel mutants.

## Introduction

The CYP enzyme family comprises 57 human isoforms serving various purposes. The human drug-metabolizing CYPs are promiscuous enzymes with broad substrate specificity transforming a variety of compounds to more soluble compounds and, thereby, facilitating their excretion from the human organism [1]. The human CYPs also comprise highly selective enzymes involved in e.g. steroidogenesis [2]. Recently, these CYPs have been shown to be potential targets for treatment of various forms of cancer [3, 4]. The plant kingdom contains 127 CYP families typically with more than 250 CYPs in each, and each CYP usually being responsible for the stereoselective synthesis of a single compound [5]. The bacterial CYPs are interesting as targets for certain diseases (e.g. *Mycobacterium tuberculosis*) [6]. Furthermore, they can be tailored to mimic human CYPs and often be expressed in higher yield than their human analogues [7].

**Fig 1:**
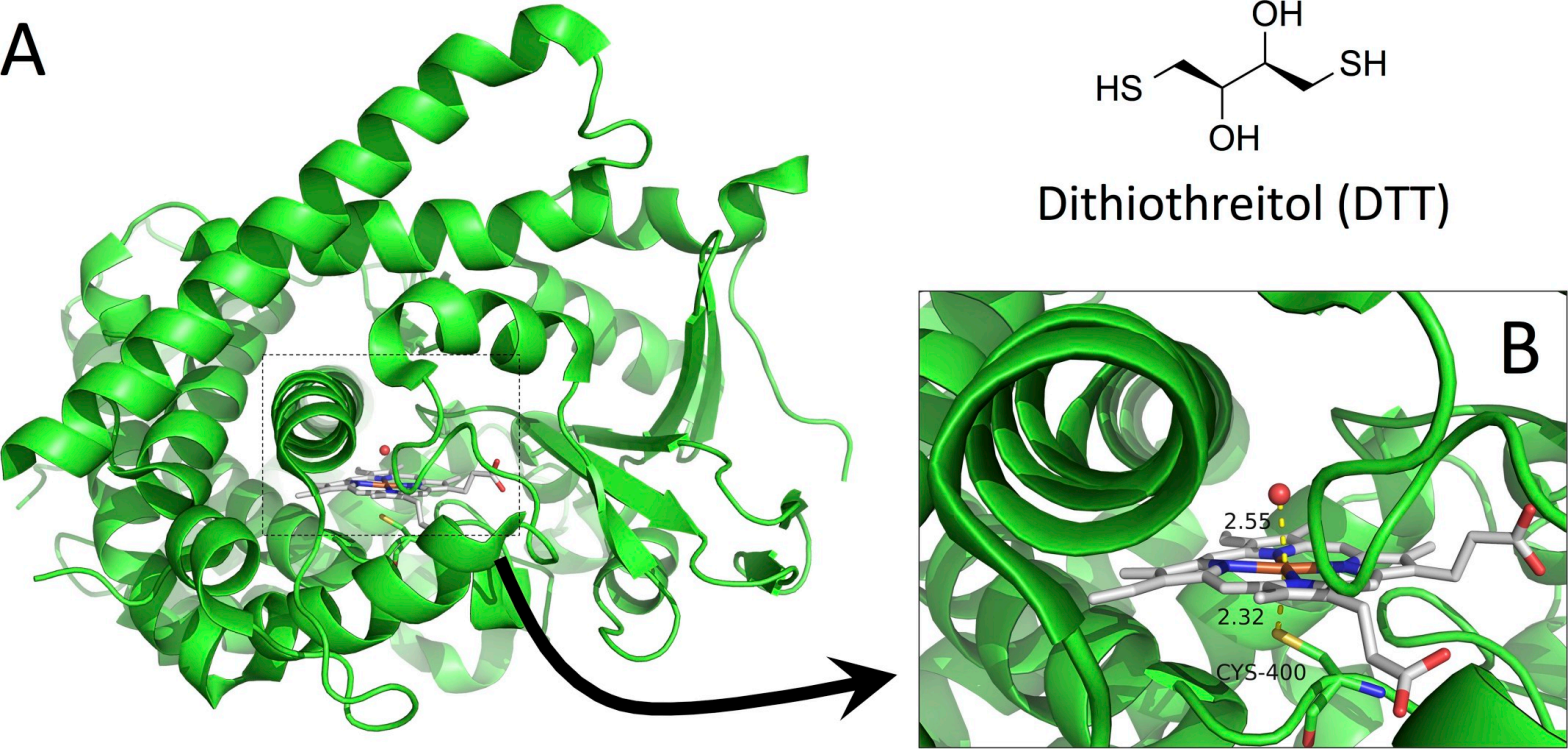
Structure of M11. (A) Three-dimensional structure of the heme domain of the CYP BM3 M11 mutant (without organic ligand in the active site; PDB entry 5E9Z) [16]. (B) Zoom-in on active site showing a water molecule and Cys400 adopting the axial positions at the hexa-coordinated Fe atom in the heme group. The 2D structure of DTT is also depicted.

CYP BM3 from *Bacillus megaterium* (CYP102A1) is characterized by high turnover, and by mutation of the natural variant it has been transformed to a biotechnologically important enzyme capable of regio- and/or stereoselective synthesis of a variety of organic compounds, including human drug metabolites [8]. A considerable part of the work by Frances H. Arnold, who was awarded the Nobel Prize 2018 in chemistry, has focused on directed evolution of the CYP BM3 to perform specific chemical reactions [9]. Several CYP BM3 mutants have been screened for their ability to metabolize drug compounds, see for example reference [10]. One mutant, the M11 mutant with mutations R47L/E64G/F81I/F87V/E143G/L188Q/Y198C/E267V/ H285Y/G415S has shown to be highly active and able to metabolize a diverse set of drug compounds [10–13]. In biocatalyst design the M11 mutant has served as template of further BM3 variants. For example, introduction of a single mutation in M11 can yield mutants that are capable of selective hydroxylation of steroids [14] or production of a toxicologically relevant metabolite of the antibiotic flucloxacillin [15]. Recently, we reported a crystal structure of this "humanized" mutant in the absence of any organic ligand in the active site (Fig 1) [16].

To analyze the possible effect of ligand binding to the promiscuous BM3 M11 variant, we present here the crystal structure of the M11 mutant of CYP BM3 in complex with dithiothreitol (DTT, Fig 1). Our crystal structure shows that DTT binds in an orientation allowing a sulfur atom to come in proximity of the heme iron and it represents the first CYP BM3 mutant M11 structure in complex with a ligand as well as the first CYP structure with a hexa-coordinated Fe atom with a mercapto-containing ligand occupying the axial position. Furthermore, we support our crystallographic study by UV/Vis spectroscopy, molecular dynamics (MD) simulations and database searches.

## Materials and methods

### Expression and purification

The M11 heme domain construct was generated as described previously [16]. In short, a stop codon was introduced after residue 473 by PCR using the following primers: FW 5’- cgcaccatgggatccATGACAATTAAAGAAATGCCTCAG-3’ and RV 5’-aaagaattcctactaTTTGCGTACTTTTTTAGCAGACTG-3’. 1 ng of pBluescript II KS (+) plasmid encoding the BM3 M11 gene was used as a template [17]. The PCR fragment was transferred to pET-28a(+) using *BamHI* and *EcoRI* restriction sites. The P450 BM3 mutant was expressed in *Escherichia coli* BL21 cells and purified using a His-tag affinity column as reported previously [18]. CYP concentrations were determined using carbon monoxide (CO) difference spectrum assay as previously described [19]. Samples showed near complete conversion to the P450 form, with negligible formation of a 420-nm peak.

To obtain the desired purity for crystallization, the protein was loaded onto a Q-Sepharose anion exchange column (16x10 cm, using the AKTA purifier) and eluted with a gradient of 0–500 mM KCl in 50 mM Tris, 1 mM EDTA, pH 7.2. Heme-domain containing fractions were then loaded onto a hydroxyapatite column (Bioscale Mini Ceramic Hydroxyapatite Cardridge, using the AKTA purifier) and eluted in a linear gradient of 25–500 mM KPi, pH 7.0 (200 ml). Pure fractions were pooled and concentrated using a 10K vivaspin membrane and the buffer was exchanged to 10 mM Tris pH 7.0 with 10% glycerol (glycerol was added for stability during storage at -20°C). The protein sample was mixed with loading dye (25mM Tris-HCl pH 6.8; 2% β-mercaptoethanol; 5% glycerol; 0.008% Bromophenol Blue; 0.8% SDS; 4 M Urea) and analyzed on SDS-PAGE. Furthermore, the sample was heated for 10 minutes at 80°C instead of 5 minutes at 95°C. Afterwards, one band of pure M11 heme construct was clearly seen. Purity of the samples was determined to be >95% using SDS-PAGE.

### Crystallization of the CYP BM3 M11 heme domain in complex with DTT

Mefenamic acid was added as solid compound to the concentrated M11 protein sample (26.2 mg/ml in 10 mM Tris-HCl buffer pH 7 with 10% glycerol) and left to equilibrate for 48 hours. Prior to the crystallization setup, DTT was added to the M11 protein sample with mefenamic acid to a final concentration of 3 mM DTT. Crystals were grown using hanging drops mixing 1 μL of protein-ligand solution with 1 μL of the reservoir solution containing 18% PEG 4000, 0.1 M Tris pH 8.0, 0.25 M MgCl_2_, and 10 mM DTT at temperature 6°C. Crystals grew within one month. The crystals were cryo-protected using reservoir solution containing 10% PEG 400 before flash-cooling in liquid nitrogen.

### Data collection, structure solution and refinement

Data collection was performed at Maxlab I911-3 [20]. The crystals belonged to space group C2 with four molecules in the asymmetric unit. The diffraction data were processed with XDS [21] and scaled using SCALA [22] in the CCP4 program suite [23]. The structure was solved by molecular replacement using PHASER [24] in CCP4 and with the structure of Cytochrome P450 BM3 (PDB ID 3DGI, mol A) [25] as the search model. The PHENIX auto-building procedure [26] was used to aid construction of the initial model of the M11 mutant-ligand complex. More than two third of the residues were rebuilt by the automatic procedure and most of the remaining residues were added manually using COOT [27]. Some loop regions in chains C and D could not be modelled due to missing electron density, as well as most of the residues of the N-terminal tags and the last approximately 15 residues of the C-terms. Residues included close to the terminals and close to the missing loops were modelled even though the electron density is weak. The structure revealed electron density close to the heme group corresponding to DTT. The final model was refined in Phenix with individual isotropic B values, TLS and riding hydrogen atoms. MolProbity [28] was used to validate the model. Refinement statistics are summarized in Table 1. The structure coordinates and corresponding structure factor file of CYP BM3 M11 mutant in complex with DTT has been deposited in the Protein Data Bank under the accession code 6IAO.

**Table 1:** Crystal data, data collection and structure refinement of CYP BM3 M11 mutant in complex with DTT.

**Data collection**	
Wavelength (Å)	1.0000
Space group	*C*2
Cell dimensions	
*a*, *b*, *c* (Å)	377.9, 59.9, 95.5
*α*, *β*, *γ* (deg.)	90.0, 95.7, 90.0
No. in asymmetric unit	4
Resolution (Å)	47.9–2.16 (2.28–2.16)
No. unique reflections	114,389 (16,259)
*R*_merge_ (%)	13.9 (72.7)
*I*/ σ*I*	4.2 (0.9)
Completeness (%)	99.6 (97.6)
Redundancy	5.1 (3.9)
CC1/2	0.99 (0.63)
**Refinement**	
*R*_work_ / *R*_free_ (%)	15.9/20.2
No. residues	
Protein	1807
HEM/DTT/PEG/GOL/Cl	4/5/2/4/20
Water	1256
*B*-factors (Å^2^)	
Protein	26.7
HEM/DTT/PEG/GOL/Cl	14.8/54.8/72.4/56.5/40.9
Water	27.1
R.m.s. deviations	
Bond lengths (Å)	0.005
Bond angles (deg.)	0.71
Non-glycine residues in allowed regions of the Ramachandran plot (%)	99.9

### Binding studies

To determine dissociation constants of binding of DTT to CYP BM3 M11, binding spectra of the substrate-protein complexes were aerobically acquired as published by Venkataraman et al. [17]. DTT was dissolved in 100 mM KPi pH 7.4 and titrated in steps of 6 μM into a cuvette containing 1 mL of 100 mM KPi pH 7.4 and 1 μM BM3 M11 domain, up to a final additional volume of not more than 2% of the initial solution volume. UV/Vis difference spectra were obtained on a Perkin Elmer Lambda 40 spectrophotometer at room temperature. The substrate-free sample was subtracted from all acquired spectra. The difference in absorbance between 380 nm and 420 nm was plotted and analyzed by nonlinear regression, by fitting the data to the ‘one site—specific binding with hill slope’ function of GraphPad Prism 5.00 (Graphpad Software, San Diego, CA, USA) in order to obtain reported K_D_ values.

### Computational details

The Protein Preparation Wizard in Maestro Software version 11.1 was used to prepare the protein structures [29]. Bond orders were assigned, hydrogens added and all water molecules removed. The hydrogen bonding network was optimized at pH 7.0. A restrained protein minimization was performed using the OPLS-2005 force field [30] with convergence of heavy atom positions to a root-mean-square deviation of 0.30 Å. Preparation of ligands was performed with LigPrep in Maestro [29]. Possible tautomers and protonation states were generated at pH 7.0 ± 2.0. Coordinates, atom types and partial atomic charges of the heme group and the neutral and mono-anionic forms of DTT are available in the S3 File. Docking was performed with GOLD (Genetic Optimisation for Ligand Docking) program version 5.6 [31]. Proteins prepared by Protein Preparation Wizard were used without additional modifications in GOLD. The binding site was defined to be within 15 Å around the heme Fe atom (using a value of 10 or 20 Å did not affect the conclusions from our docking study). Ligands prepared by LigPrep were exported from Maestro. Ligands were docked 10 times with slow genetic algorithm and with the heme-modified ChemScore as the scoring function [32].

The systems for the MD simulations were created with the Desmond system builder using the SPC water model yielding an orthorhombic box with a buffer size of 10 Å between the protein and the box boundary. The system was neutralized with Na^+^ ions. The final systems contained a total of close to 60,000 atoms including 7,360 atoms for the protein including the heme group, 18 atoms for DTT, 13 Na^+^ ions and approximately 18,000 water molecules for the neutral forms of DTT. MD simulations were performed with the Desmond program (version 3.6) [33] using the OPLS-2005 force field [30]. The default equilibration protocol was used for equilibrating the systems. Subsequently, the systems were simulated for 100 ns.

## Results and discussion

### X-ray crystallography

**Fig 2:**
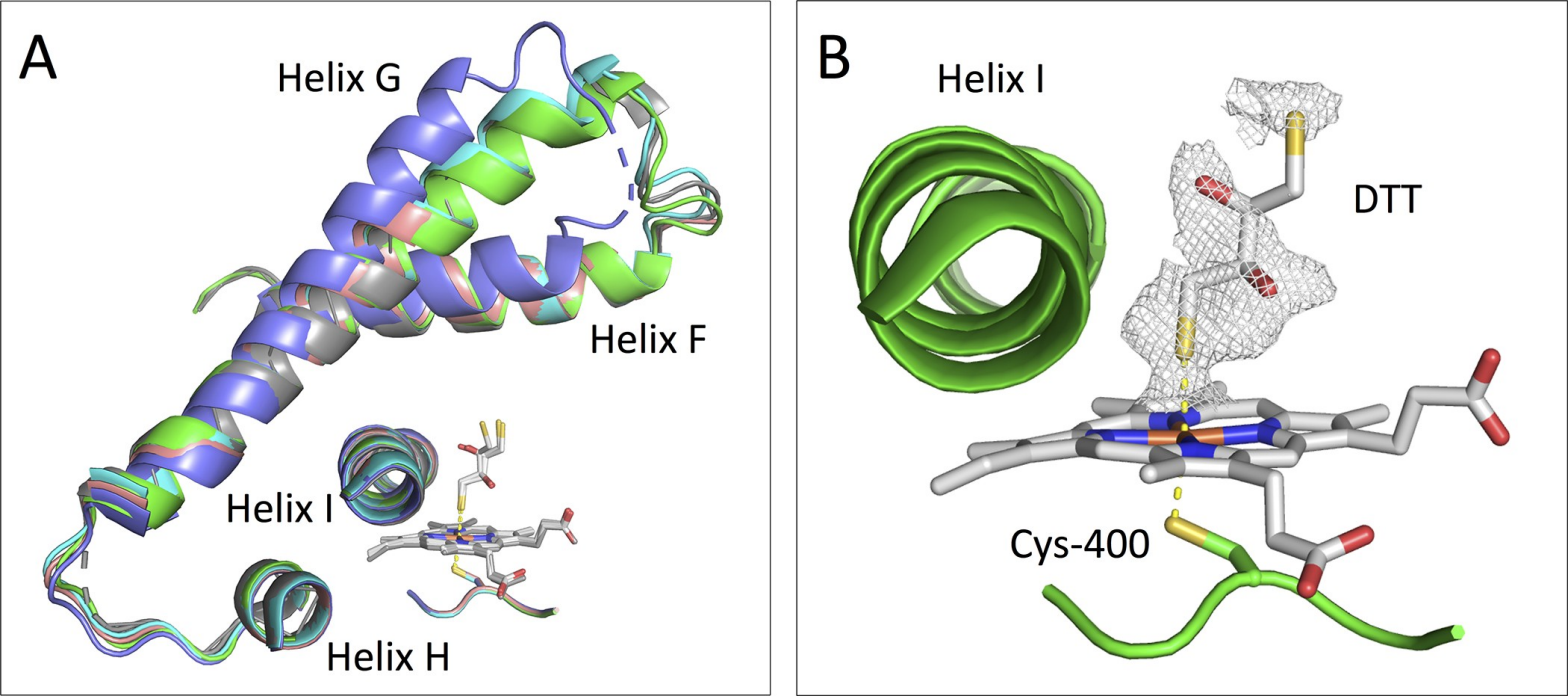
Final structure of the heme domain of the CYP BM3 M11 mutant in complex with DTT. (A) Chains B, C, and D are superposed on chain A. For comparison PDB entry 1JPZ (chain A) with the SB conformation [34] is superposed on chain A. Only helices F, G and H with the loops between them are shown as cartoon, together with helix I and Cys400. DTT and the heme moiety from all four chains are shown as sticks and colored white. The A chain is colored green, B cyan, C salmon, D slate and 1JPZ in grey. (B) Zoom on helix I and Cys400 of chain A together with its heme group and DTT, with 2Fo−Fc omit electron density map contoured at 1σ and carved at 2.0 A° around DTT.

The crystal structure of the M11 mutant of the heme domain of CYP BM3 in complex with DTT was determined and refined at a resolution of 2.16 Å (Table 1). The overall structure is similar to the previously published structure of the M11 mutant (PDB entry 5E9Z): it shows four enzyme molecules in the asymmetric unit (S1 Fig, S1 and S2 Tables) [16] and the enzyme is observed in the substrate-bound (SB) conformation [8]. The SB conformation of M11 is compared to 1JPZ [34] and the superposition shows that the access channel is closed, as helices F, G and H and the loops between them are tilted (Fig 2A). Helix I is observed with a small kink as described for the SB conformation (Fig 2). The conformations of chain C and D are difficult to describe because some loop areas were not observed, although chain D appears to adopt a slightly different conformation for helix F and G (Fig 2A).

DTT was added to the protein sample and to the reservoir solution before crystallizing the M11 protein ligand complex. As found by Capoferri et al. [16], a DTT molecule is observed to form a disulfide bridge between the Cys198 residues in chain A and B. Due to the unobserved loop regions in chain C and D, this disulfide bridge is not found here. The DTT molecule observed between chain A and B is refined with full occupancy, indicating full presence of the disulfide bridge (S2 Fig). This may be a reason for the observed crystal packing, which comprises four M11 protein chains in the asymmetric unit.

In contrast to our previous crystallographic study [16], the observed electron densities in the active sites close to the heme iron in all four chains in our current structure reveal the presence of DTT (Fig 2B, and S3 Fig). One of the sulfur atoms of DTT is observed close to the heme iron. The Fe–S_DTT_ distance is 2.32 Å in the A chain, which suggests ligand coordination as it is comparable to the interaction observed on the other side of the heme group, where the Fe–S_Cys400_ distance is 2.31 Å (Fig 2B). Note that the Fe–S distances (Fe–S_DTT_ and Fe–S_Cys400_) used for refinement are based on the considerations described in the next subsection (Related crystal structures) and a similar value for these two Fe-S distances is in line with our results described in the Spectroscopic studies subsection.

Direct hydrogen bonding interactions between the DTT molecules and active site residues or water molecules are not observed. In all four chains DTT has been modelled in a similar conformation and orientation but the electron density is not sufficiently well-defined to identify the precise positions of all DTT atoms, which may indicate ligand flexibility. Several different orientations or conformations of DTT can be modelled into the density map, with a sulfur atom coordinating Fe in all of them. Since DTT was added as a racemic mixture, both the *R*,*R*- and *S*,*S*-enantiomers have been modelled into the electron density. It cannot unambiguously be identified which enantiomer is, or if a mixture of both are coordinating Fe. Only the *R*,*R*-enantiomer is included in the final structure.

### Related crystal structures

The Protein Data Bank [35] contains more than 70 CYP BM3 structures (cf. S3 Table). The majority of these structures were determined to study the effect of single or multiple amino acid mutations on the ability of the enzyme to perform various organic reactions. DTT is only observed for the structure 5E9Z and our structure.

**Fig 3:**
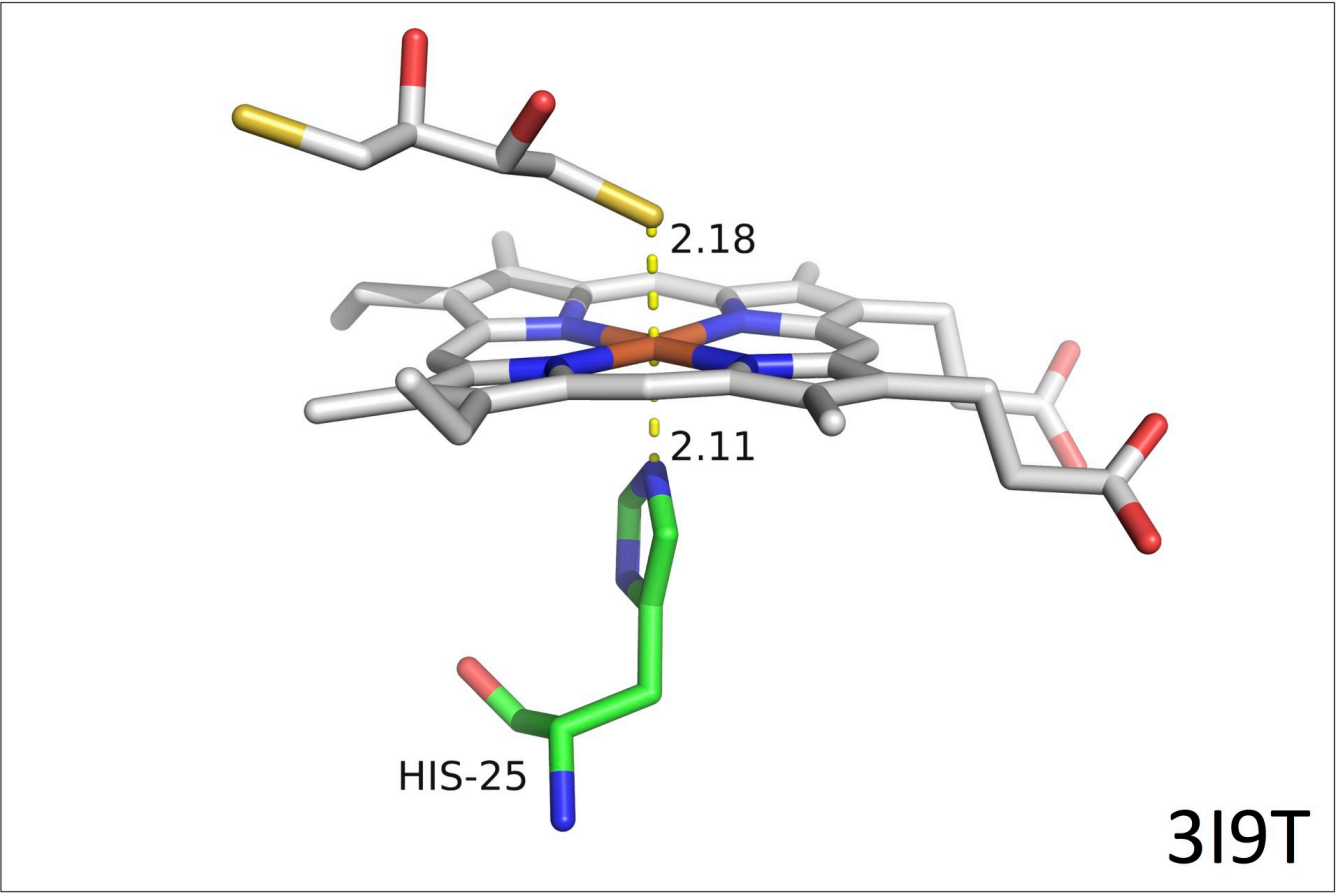
3I9T crystal structure. Heme group, His_25_ and DTT from the 3D structure of DTT binding to rat heme oxygenase (PDB entry 3I9T [36]).

In the Protein Data Bank, we identified three different types of protein structures with a mercapto-containing compound coordinating to the Fe atom in a porphyrin ring. In the first group hydrogen sulfide or a low-molecular weight mercaptane, including DTT and DTT stereoisomers, occupies the axial position as the sixth ligand coordinating the Fe atom, but in all these cases, the other axial ligand is a histidine. The shortest Fe-S distance (2.18 Å) is observed for DTT binding to rat heme oxygenase (PDB entry 3I9T, Fig 3) [36]. Slightly longer Fe-S distances (2.2–2.3 Å) are observed for the other members of this first group (PDB entries 3I8R, 3I9U, 4HPA, 4HPB, 4HPC, 4HPD and 4V2K, S4 and S5 Figs) [36–38]. A second group comprises two structures (PDB entries 2EVP and 2PBJ) with penta-coordinated Fe atoms, which show longer Fe-S distances of 2.41 and 2.65 Å, respectively (S5 Fig) [39, 40]. Finally, the third group contains only one member, i.e., the *Azotobacter vinelandii* bacterioferritin structure (PDB entry 2FKZ), where the porphyrin ring is sandwiched between two methionine residues (S5 Fig) [41].

Three-dimensional structures of heme-complexes are not only available from the Protein Data Bank (PDB). The Cambridge Structural Database (CSD) [42] contains several porphyrin structures, although a limited number of these have a sulfur atom coordinating the iron atom and only a few are relevant in our context. The advantage of the CSD structures relative to the PDB structures is generally that the hydrogens are unambiguously determined.

**Fig 4:**
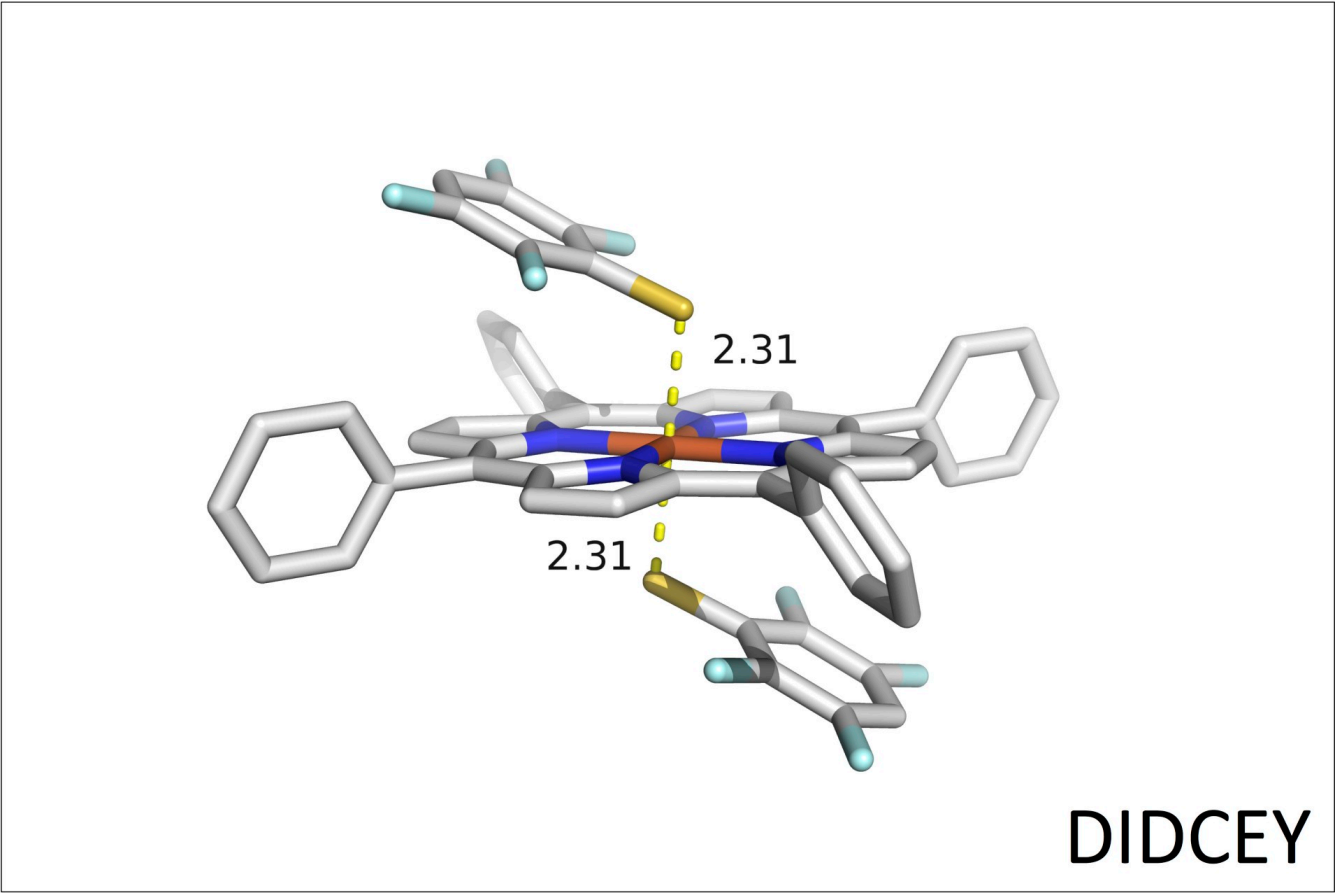
DIDCEY crystal structure. 3D representation of the DIDCEY structure containing a porphyrin ring with two 2,3,5,6-tetrafluorobenzenethiolate ions coordinated to the Fe atom [43].

The DIDCEY structure from CSD is especially interesting because the iron is hexa-coordinated with two 2,3,5,6-tetrafluorobenzenethiolate ions occupying the axial positions. The Fe-S distances are 2.31 Å and the porphyrin ring is completely planar (Fig 4) [43].

Four additional sulfur-iron coordinating heme complexes are presented in S6 Fig. These include the CORNAY structure, which has a single hydrosulfide ion (HS-) coordinating the low-spin iron(III) as the axial ligand with a Fe-S distance of 2.30 Å [44]. In the WAHLAU and WAHLEY structures a hydrosulfide ion (HS-) is coordinating the high-spin iron(II) with a Fe-S distance of 2.4 Å [45]. In JELMIW an ethanethiolate ion is taking up the axial position relative to the high-spin iron(II) atom with a Fe-S distance of 2.32 Å [46]. These four structures are characterized with non-planar porphyrin rings (cf. S6 Fig).

The above CSD structures confirm that the mercapto-compounds bind in their anionic form to the Fe atom in the porphyrin ring. Finally, the analysis of the observed Fe-S distances observed in PDB and CSD has been used as guide during structure refinement.

### Spectroscopic studies

**Fig 5:**
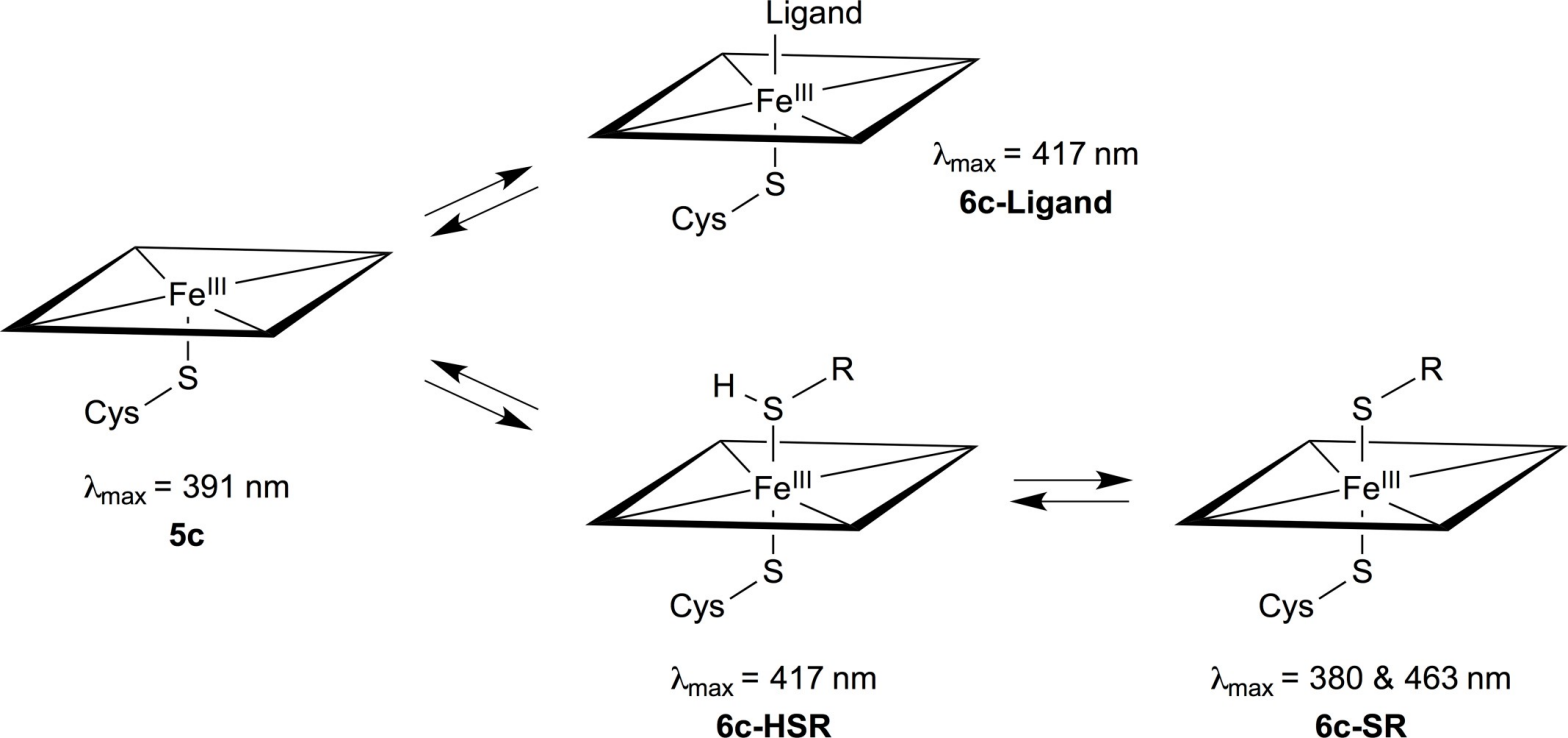
Thiol-heme binding. The equilibria between binding of thiols, thiolates and other ligands to heme proteins as suggested in reference [48]. **5c** and **6c** refer to the iron being five- and six-coordinated, respectively. Soret maxima are from reference [48] as well.

Binding of sulfur-containing compounds to heme proteins has been studied by spectroscopic methods by several researchers [47–49]. Based on UV/Vis spectroscopy, magnetic circular dichroism and electron paramagnetic resonance spectroscopy, Sono et al. showed some time ago that binding of thiols and thiolates to ferric Cytochrome P450cam is competitive with substrate binding and that thiolates typically bind three order of magnitudes better than thiols [48]. From the observed Soret maxima of the P450cam-thiol(ate) complexes they also concluded that the sixth sulfur ligand was binding as a mixture of the thiol (**6c-HSR**) and thiolate (**6c-SR**), and that the equilibrium between the thiol-thiolate complexes was pH dependent (Fig 5). Finally, they showed that the pK_a_ values of the thiols might be lowered with up to four units by ligation to P450cam [48].

In a recent publication, Sono et al. extended their original work to ferrous heme-containing proteins [49]. By UV/Vis spectroscopy they showed that ethanethiol binds to the heme in its anionic thiolate form. Gorren et al. [47] studied the binding of DTT and other sulfur-containing ligands, to neuronal nitric oxide synthase (nNOS). They found that DTT binds to the heme group and forms a bisthiolate complex with an apparent dissociation constant K_D_ = 0.16 nM. DTT was also shown to be a L-arginine competitive inhibitor with K_i_ = 11 mM.

**Fig 6:**
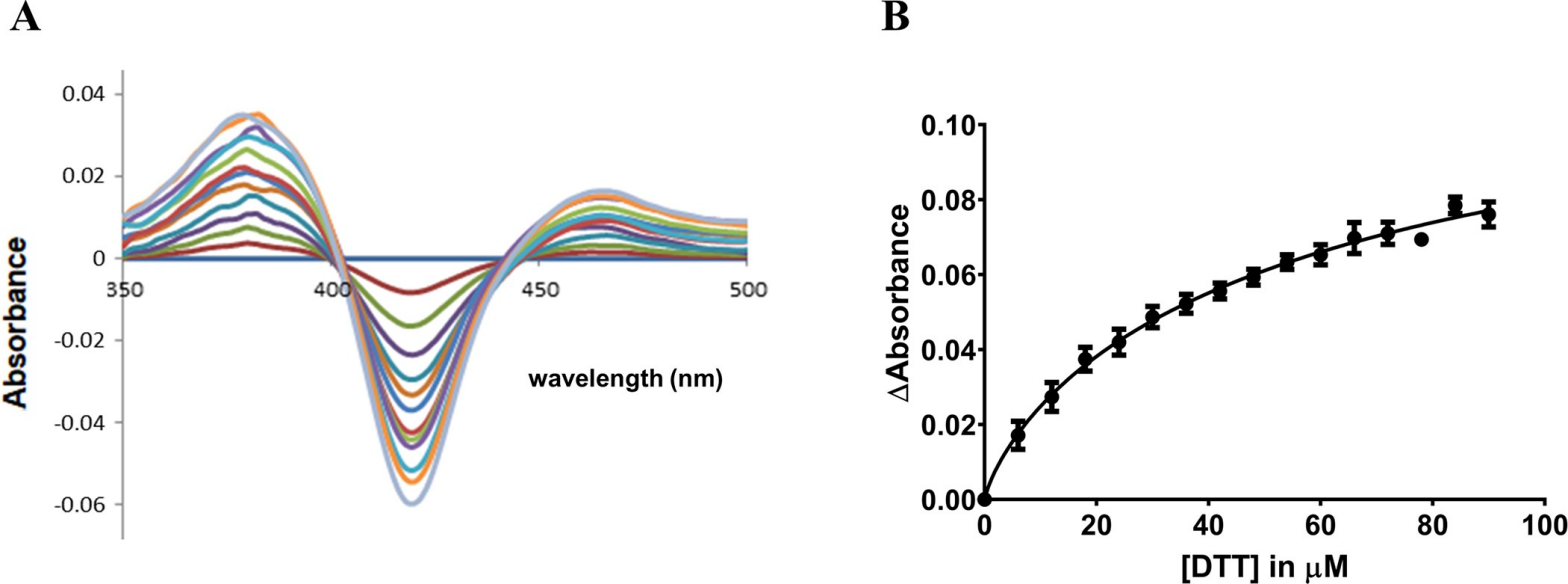
DTT to M11 binding. (A) UV/Vis absorbance difference spectra for binding of DTT to CYP BM3 mutant M11 at different DTT concentrations between 0 and 96 μM (increasing in steps of 6 μM, as indicated by the different lines with the dark-brown line corresponding to 6 μM and the lightblue line to 96 μM). (B) Differences in absorbance between 380 and 420 nm at the different DTT concentrations as used for fitting apparent values for dissociation constant K_D_. Averages are given for triplicated experiments, together with associated error bars.

Here we performed UV/Vis spectrophotometric titration studies of CYP BM3 M11 with DTT to study its type of binding (Fig 6). The obtained absorbance difference spectra show maxima at approximately 380 and 463 nm, and a minimum close to 417 nm (*i*.*e*., at 420 nm, Fig 6A). From a comparison with the Soret maxima reported by Sono et al. in their studies on binding of sulfur-containing compounds to P450cam [48], this confirms the possibility of heme iron coordination by DTT as observed in our crystal structure and it indicates that DTT coordinates in its anionic form to Fe (cf. Fig 5). The apparent K_D_ value was fitted from Fig 6B, and determined to be 56.8 ± 1.3 μM.

### Molecular dynamics simulations

**Fig 7:**
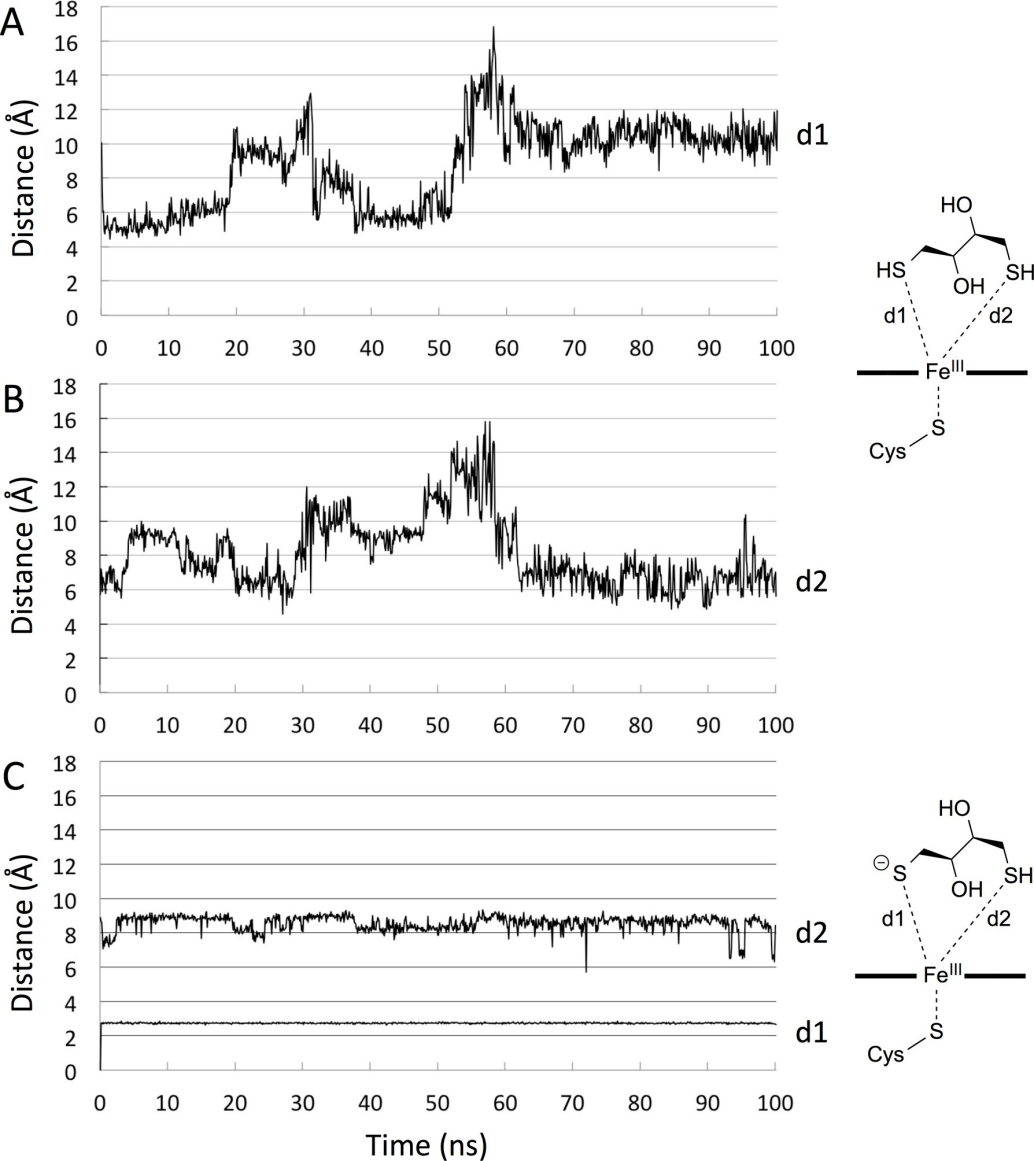
Fe-S distances from MD. Time series of Fe-S distances from MD simulations of CYP BM3 mutant M11 in complex with the neutral form (A and B) and monovalent anion (C) of the (*R*,*R*)-isomer of DTT.

The DTT molecule, both in the neutral form (both as *R*,*R*- and *S*,*S*-isomer) and as the monovalent anion (only *R*,*R*-isomer), was docked into the active site of the CYP BM3 M11 mutant using the GOLD docking program and the ChemScore scoring function developed specifically for docking to CYPs [31,32]. For the neutral forms of DTT, we obtained several binding poses, and the best scoring poses were positioned with one of the DTT sulfur atom in the vicinity of the Fe atom (2.4–2.6 Å). Four poses were selected as input for 100 ns of MD simulations. Only the results of the MD simulation for the pose with the shortest Fe-S distance will be discussed here. During the MD simulation the Fe-S distances d1 and d2 varied considerably (d1 = 8.1 ± 2.0 Å and d2 = 8.7 ± 2.5 Å, cf. Fig 7) and none of the sulfur atoms got closer to Fe than 5 Å after equilibration. Actually, it appeared as the neutral DTT molecule slowly drifted away from the heme group in all simulations.

In contrast, docking of the mono-anion form of *R*,*R*-isomer of DTT only yielded a single pose positioned with the negatively charged sulfur atom coordinating directly to the Fe atom (2.61 atom vicinity teractionsgroup, probabthe heme group. dons Å). MD simulations with this pose as starting conformation revealed that the DTT mono-anion maintained its contact with Fe (d1 = 2.74 ± 0.03 Å and d2 = 8.56 ± 0.47 Å) (Fig 7).

Thus, the MD simulations confirm close contacts between the Fe and the negatively charged sulfur, and that there are no specific interactions between the remaining of the DTT molecule and the protein residues.

## Conclusions

We presented a crystal structure of CYP BM3 mutant M11 in complex with a ligand. Over the last 10 years, this “humanized” CYP BM3 variant has shown potential as biocatalyst and as template to engineer mutants that selectively metabolize drugs and drug-like compounds. Our crystal structure represents, to our knowledge, the first structure of the CYP BM3 mutant M11 with a ligand, DTT, bound directly to the heme iron. Our crystal structure indicates that binding of a DTT ligand to M11 does not significantly alter the protein conformation, which underlines the use of the structure of this promiscuous enzyme for modeling purposes. Our work also shows that DTT coordinates with the heme iron, with a Fe-S distance of 2.3 Å. Absorbance difference spectra obtained in UV/Vis spectroscopic titration of M11 with DTT demonstrate coordination by the ligand in its anionic form. Docking and molecular dynamics simulations also suggest that the monovalent DTT anion can adopt a heme iron-coordinating binding pose, in which no specific interactions between DTT and the active-site residues are observed. This finding is in line with our interpretation from the obtained electron density map that DTT remains relatively flexible in its observed active-site bound conformation.

## Supporting information

S1 Fig Comparison between the structure of the CYP BM3 M11 mutant in complex with DTT and the M11 structure without organic ligand (PDB entry 5E9Z). Protein chains are shown as cartoons with the heme group and DTT in sticks. DTT-bound M11 is colored green and 5E9Z in pale cyan. Root-mean-square deviations are presented in S1 Table. (A) Chain A from both structures. (B) Chain B from both structures. (C) Chain C from both structures. (D) Chain D from both structures.
(PDF)

S2 Fig The DTT molecule bridging the A and B chain in the CYP BM3 M11 structure. Potential H-bonds between DTT and Arg190 and three water molecules are shown as dashed black lines. Part of the protein is shown as cartoon (chain A in green and chain B in cyan), DTT and Arg190 in sticks.
(PDF)

S3 Fig Structure of CYP450 BM3 M11 in complex with DTT. The heme binding site with DTT is shown with 2Fo−Fc omit electron density maps contoured at 1σ and carved at 2.0 A° around DTT. Helices F, G and I are shown in cartoon representation and DTT, heme group and Cys400 in stick representation. (A) Chain A. (B) Chain B. (C) Chain C. (D) Chain D.
(PDF)

S4 Fig Structures of mercapto-containing ligands coordinating to the Fe atom in a porphyrin group. PDB entry 3I8R, chains A, B and C; and PDB entry 3I9U.
(PDF)

S5 Fig Structures from the Protein Data Bank of sulfur-containing ligands coordinating to the Fe atom in a porphyrin group. PDB entries 4HPA, 4HPB, 4HPC, 4HPD, 4V2K, 2EVP, 2PBJ, and 2FKZ.
(PDF)

S6 Fig Structures from the Cambridge Structural Database containing a porphyrin ring with axial sulfur-containing compounds. CORNAY, JELMIW, WAHLAU, and WAHLEY.
(PDF)

S1 Table The present structure of CYP BM3 M11 in complex with DTT, compared to the structure without ligand (5E9Z). Root-mean-square deviations (RMSDs, in Å) between chains. Alignment is performed and RMSDs are calculated for C_α_ atoms using Pymol (Version 2.0.6, Schrodinger). Structures are shown in S1 Fig.
(PDF)

S2 Table Root-mean-square deviations (RMSDs, in Å) between the four protein chains in the asymmetric unit of the present CYP BM3 M11 structure. Chain A was used for comparison. Alignment is performed and RMSDs are calculated for C_α_ atoms using Pymol (Version 2.0.6, Schrodinger).
(PDF)

S3 Table Cytochrome P450 BM3 structures from PDB. Literature references in S1 File.
(PDF)

S1 File Literature references. References used in S6 Fig and S3 Table.
(PDF)

S2 File Validation report. Copy of the PDB X-ray Structure Validation Report.
(PDF)

S3 File Force field parameters and coordinates. Atomic coordinates, atom types and partial atomic charges of neutral and anionic DTT and the heme group.
(PDF)

## References

[1] Ortiz de Montellano PR. Cytochrome P450: Structure, Mechanism and Biochemistry. 4 ed New York: Springer International Publishing; 2015.

[2] Nebert DW, Wikvall K, Miller WL. Human cytochromes P450 in health and disease. Philos Trans R Soc Lond B Biol Sci. 2013;368(1612):20120431 10.1098/rstb.2012.0431 23297354 PMC3538421

[3] Swanson HI, Njar VCO, Yu Z, Castro DJ, Gonzalez FJ, Williams DE, et al Targeting drug-metabolizing enzymes for effective chemoprevention and chemotherapy. Drug Metab Dispos. 2010;38(4):539–44. 10.1124/dmd.109.031351 20233842 PMC2845935

[4] Bonomo S, Hansen CH, Petrunak EM, Scott EE, Styrishave B, Jørgensen FS, et al Promising Tools in Prostate Cancer Research: Selective Non-Steroidal Cytochrome P450 17A1 Inhibitors. Sci Rep. 2016;6:29468 10.1038/srep29468 27406023 PMC4942611

[5] Hamberger B, Bak S. Plant P450s as versatile drivers for evolution of species-specific chemical diversity. Philos Trans R Soc Lond B Biol Sci. 2013;368(1612):20120426 10.1098/rstb.2012.0426 23297350 PMC3538417

[6] Hudson SA, McLean KJ, Munro AW, Abell C. Mycobacterium tuberculosis cytochrome P450 enzymes: a cohort of novel TB drug targets. Biochem Soc Trans. 2012;40(3):573–9. 10.1042/BST20120062 22616869

[7] Yun CH, Kim KH, Kim DH, Jung HC, Pan JG. The bacterial P450 BM3: a prototype for a biocatalyst with human P450 activities. Trends Biotechnol. 2007;25(7):289–98. 10.1016/j.tibtech.2007.05.003 17532492

[8] Whitehouse CJ, Bell SG, Wong LL. P450(BM3) (CYP102A1): connecting the dots. Chem Soc Rev. 2012;41(3):1218–60. 10.1039/c1cs15192d 22008827

[9] Arnold FH. Directed Evolution: Bringing New Chemistry to Life. Angew Chem Int Ed Engl. 2018;57(16):4143–8. 10.1002/anie.201708408 29064156 PMC5901037

[10] Reinen J, van Leeuwen JS, Li Y, Sun L, Grootenhuis PD, Decker CJ, et al Efficient screening of cytochrome P450 BM3 mutants for their metabolic activity and diversity toward a wide set of drug-like molecules in chemical space. Drug Metab Dispos. 2011;39(9):1568–76. 10.1124/dmd.111.039461 21673132

[11] van Vugt-Lussenburg BM, Stjernschantz E, Lastdrager J, Oostenbrink C, Vermeulen NP, Commandeur JN. Identification of critical residues in novel drug metabolizing mutants of cytochrome P450 BM3 using random mutagenesis. J Med Chem. 2007;50(3):455–61. 10.1021/jm0609061 17266197

[12] Damsten MC, van Vugt-Lussenburg BM, Zeldenthuis T, de Vlieger JS, Commandeur JN, Vermeulen NP. Application of drug metabolising mutants of cytochrome P450 BM3 (CYP102A1) as biocatalysts for the generation of reactive metabolites. Chem Biol Interact. 2008;171(1):96–107. 10.1016/j.cbi.2007.09.007 17996858

[13] Boerma JS, Vermeulen NP, Commandeur JN. Application of CYP102A1M11H as a tool for the generation of protein adducts of reactive drug metabolites. Chem Res Toxicol. 2011;24(8):1263–74. 10.1021/tx2001515 21639118

[14] Rea V, Kolkman AJ, Vottero E, Stronks EJ, Ampt KAM, Honing M, et al Active Site Substitution A82W Improves the Regioselectivity of Steroid Hydroxylation by Cytochrome P450 BM3 Mutants As Rationalized by Spin Relaxation Nuclear Magnetic Resonance Studies. Biochemistry. 2012;51(3):750–60. 10.1021/bi201433h 22208729

[15] Luirink RA, Dekker SJ, Capoferri L, Janssen LFH, Kuiper CL, Ari ME, et al A combined computational and experimental study on selective flucloxacillin hydroxylation by cytochrome P450 BM3 variants. J Inorg Biochem. 2018;184:115–22. 10.1016/j.jinorgbio.2018.04.013 29723739

[16] Capoferri L, Leth R, ter Haar E, Mohanty AK, Grootenhuis PD, Vottero E, et al Insights into regioselective metabolism of mefenamic acid by cytochrome P450 BM3 mutants through crystallography, docking, molecular dynamics, and free energy calculations. Proteins. 2016;84(3):383–96. 10.1002/prot.24985 26757175

[17] Venkataraman H, de Beer SBA, Geerke DP, Vermeulen NPE, Commandeur JNM. Regio- and Stereoselective Hydroxylation of Optically Active alpha-Ionone Enantiomers by Engineered Cytochrome P450 BM3 Mutants. Adv Synth Catal. 2012;354(11–12):2172–84.

[18] Vottero E, Rea V, Lastdrager J, Honing M, Vermeulen NP, Commandeur JN. Role of residue 87 in substrate selectivity and regioselectivity of drug-metabolizing cytochrome P450 CYP102A1 M11. J Biol Inorg Chem. 2011;16(6):899–912. 10.1007/s00775-011-0789-4 21567268 PMC3139092

[19] Omura T, Sato R. The Carbon Monoxide-Binding Pigment of Liver Microsomes. I. Evidence for Its Hemoprotein Nature. J Biol Chem. 1964;239:2370–8. 14209971

[20] Ursby T, Unge J, Appio R, Logan DT, Fredslund F, Svensson C, et al The macromolecular crystallography beamline I911-3 at the MAX IV laboratory. J Synchrotron Radiat. 2013;20(Pt 4):648–53. 10.1107/S0909049513011734 23765310 PMC3943556

[21] Kabsch W. Xds. Acta Crystallogr D Biol Crystallogr. 2010;66(Pt 2):125–32. 10.1107/S0907444909047337 20124692 PMC2815665

[22] Evans PR. An introduction to data reduction: space-group determination, scaling and intensity statistics. Acta Crystallogr D Biol Crystallogr. 2011;67(Pt 4):282–92. 10.1107/S090744491003982X 21460446 PMC3069743

[23] Winn MD, Ballard CC, Cowtan KD, Dodson EJ, Emsley P, Evans PR, et al Overview of the CCP4 suite and current developments. Acta Crystallogr D Biol Crystallogr. 2011;67(Pt 4):235–42. 10.1107/S0907444910045749 21460441 PMC3069738

[24] McCoy AJ, Grosse-Kunstleve RW, Adams PD, Winn MD, Storoni LC, Read RJ. Phaser crystallographic software. J Appl Crystallogr. 2007;40(Pt 4):658–74. 10.1107/S0021889807021206 19461840 PMC2483472

[25] Roberts AG, Katayama J, Kaspera R, Ledwitch KV, Le Trong I, Stenkamp RE, et al The role of cytochrome P450 BM3 phenylalanine-87 and threonine-268 in binding organic hydroperoxides. Biochim Biophys Acta. 2016;1860(4):669–77. 10.1016/j.bbagen.2015.12.014 26723172

[26] Adams PD, Afonine PV, Bunkoczi G, Chen VB, Davis IW, Echols N, et al PHENIX: a comprehensive Python-based system for macromolecular structure solution. Acta Crystallogr D Biol Crystallogr. 2010;66(Pt 2):213–21. 10.1107/S0907444909052925 20124702 PMC2815670

[27] Emsley P, Lohkamp B, Scott WG, Cowtan K. Features and development of Coot. Acta Crystallogr D Biol Crystallogr. 2010;66(Pt 4):486–501. 10.1107/S0907444910007493 20383002 PMC2852313

[28] Chen VB, Arendall WB 3rd, Headd JJ, Keedy DA, Immormino RM, Kapral GJ, et al MolProbity: all-atom structure validation for macromolecular crystallography. Acta Crystallogr D Biol Crystallogr. 2010;66(Pt 1):12–21. 10.1107/S0907444909042073 20057044 PMC2803126

[29] Sastry GM, Adzhigirey M, Day T, Annabhimoju R, Sherman W. Protein and ligand preparation: parameters, protocols, and influence on virtual screening enrichments. J Comput Aided Mol Des. 2013;27(3):221–34. 10.1007/s10822-013-9644-8 23579614

[30] Banks JL, Beard HS, Cao Y, Cho AE, Damm W, Farid R, et al Integrated Modeling Program, Applied Chemical Theory (IMPACT). J Comput Chem. 2005;26(16):1752–80. 10.1002/jcc.20292 16211539 PMC2742605

[31] Jones G, Willett P, Glen RC, Leach AR, Taylor R. Development and validation of a genetic algorithm for flexible docking. J Mol Biol. 1997;267(3):727–48. 10.1006/jmbi.1996.0897 9126849

[32] Kirton SB, Murray CW, Verdonk ML, Taylor RD. Prediction of binding modes for ligands in the cytochromes P450 and other heme-containing proteins. Proteins. 2005;58(4):836–44. 10.1002/prot.20389 15651036

[33] Desmond Molecular Dynamics System. D.E. Shaw Research, New York, NY 2009.

[34] Haines DC, Tomchick DR, Machius M, Peterson JA. Pivotal role of water in the mechanism of P450BM-3. Biochemistry. 2001;40(10):13456–65. 11695892 10.1021/bi011197q

[35] Berman HM, Westbrook J, Feng Z, Gilliland G, Bhat TN, Weissig H, et al The Protein Data Bank. Nucleic Acids Res. 2000;28(1):235–42. 10.1093/nar/28.1.235 10592235 PMC102472

[36] Matsui T, Iwasaki M, Sugiyama R, Unno M, Ikeda-Saito M. Dioxygen Activation for the Self-Degradation of Heme: Reaction Mechanism and Regulation of Heme Oxygenase. Inorg Chem. 2010;49(8):3602–9. 10.1021/ic901869t 20380462

[37] He C, Nishikawa K, Erdem ÖF, Reijerse E, Ogata H, Lubitz W, et al Complexes of ferriheme nitrophorin 4 with low-molecular weight thiol(ate)s occurring in blood plasma. J Inorg Biochem. 2013;122:38–48. 10.1016/j.jinorgbio.2013.01.012 23474537

[38] Grabarczyk DB, Chappell PE, Eisel B, Johnson S, Lea SM, Berks BC. Mechanism of thiosulfate oxidation in the SoxA family of cysteine-ligated cytochromes. J Biol Chem. 2015;290(14):9209–21. 10.1074/jbc.M114.618025 25673696 PMC4423706

[39] Qin J, Perera R, Lovelace LL, Dawson JH, Lebioda L. Structures of Thiolate- and Carboxylate-Ligated Ferric H93G Myoglobin: Models for Cytochrome P450 and for Oxyanion-Bound Heme Proteins. Biochemistry. 2006;45(10):3170–7. 10.1021/bi052171s 16519512 PMC2556877

[40] Yamada T, Takusagawa F. PGH2 Degradation Pathway Catalyzed by GSH−Heme Complex Bound Microsomal Prostaglandin E2 Synthase Type 2: The First Example of a Dual-Function Enzyme. Biochemistry. 2007;46(28):8414–24. 10.1021/bi700605m 17585783

[41] Swartz L, Kuchinskas M, Li H, Poulos TL, Lanzilotta WN. Redox-Dependent Structural Changes in the Azotobacter vinelandii Bacterioferritin: New Insights into the Ferroxidase and Iron Transport Mechanism. Biochemistry. 2006;45(14):4421–8. 10.1021/bi060146w 16584178

[42] Groom CR, Bruno IJ, Lightfoot MP, Ward SC. The Cambridge Structural Database. Acta Crystallogr B. 2016;72(2):171–9. 10.1107/S2052520616003954 PMC4822653 27048719

[43] Doppelt P, Fischer J, Weiss R. Synthesis and Structure of a Dimercapto—Iron(III) Porphyrin Derivative: | Fe(SC6HF4)2TPP | | Na c 18C6|, C6H6. Croat Chem Acta. 1984;57:507–18.

[44] English DR, Hendrickson DN, Suslick KS, Eigenbrot CW, Scheidt WR. Low-spin five-coordinate ferric porphyrin complex: [5, 10, 15, 20-tetrakis(4-methoxyphenyl)porphyrinato] (hydrosulfido)iron(III). J Am Chem Soc. 1984;106(23):7258–9.

[45] Pavlik JW, Noll BC, Oliver AG, Schulz CE, Scheidt WR. Hydrosulfide (HS−) Coordination in Iron Porphyrinates. Inorg Chem. 2010;49(3):1017–26. 10.1021/ic901853p 20038134 PMC2811220

[46] Schappacher M, Ricard L, Fischer J, Weiss R, Montiel-Montoya R, Bill E, et al Synthesis, structure, and spectroscopic properties of five-coordinate mercaptoiron(II) porphyrins. Models for the reduced state of cytochrome P450. Inorg Chem. 1989;28(26):4639–45.

[47] Gorren ACF, Schrammel A, Schmidt K, Mayer B. Thiols and Neuronal Nitric Oxide Synthase: Complex Formation, Competitive Inhibition, and Enzyme Stabilization. Biochemistry. 1997;36(14):4360–6. 10.1021/bi962381s 9100033

[48] Sono M, Andersson LA, Dawson JH. Sulfur donor ligand binding to ferric cytochrome P-450-CAM and myoglobin. Ultraviolet-visible absorption, magnetic circular dichroism, and electron paramagnetic resonance spectroscopic investigation of the complexes. J Biol Chem. 1982;257(14):8308–20. 6282878

[49] Sono M, Sun S, Modi A, Hargrove MS, Molitor B, Frankenberg-Dinkel N, et al Spectroscopic evidence supporting neutral thiol ligation to ferrous heme iron. J Biol Inorg Chem. 2018;23(7):1085–92. 10.1007/s00775-018-1611-3 30251130

