# Supplementary material for: Structural analysis of Cytochrome P450 BM3 mutant M11 in complex with dithiothreitol
Source: PLoS One. 2019 May 24;14(5):e0217292. doi: 10.1371/journal.pone.0217292 (PMC6534296; doi:10.1371/journal.pone.0217292)
Supplement: S1 Fig — Protein chains are shown as cartoons with the heme group and DTT in sticks. DTT-bound M11 is colored green and 5E9Z in pale cyan. Root-mean-square deviations are presented in S1 Table. (A) Chain A from both structures. (B) Chain B from both structures. (C) Chain C from both structures. (D) Chain D from both structures. (PDF) [file pone.0217292.s001.pdf]

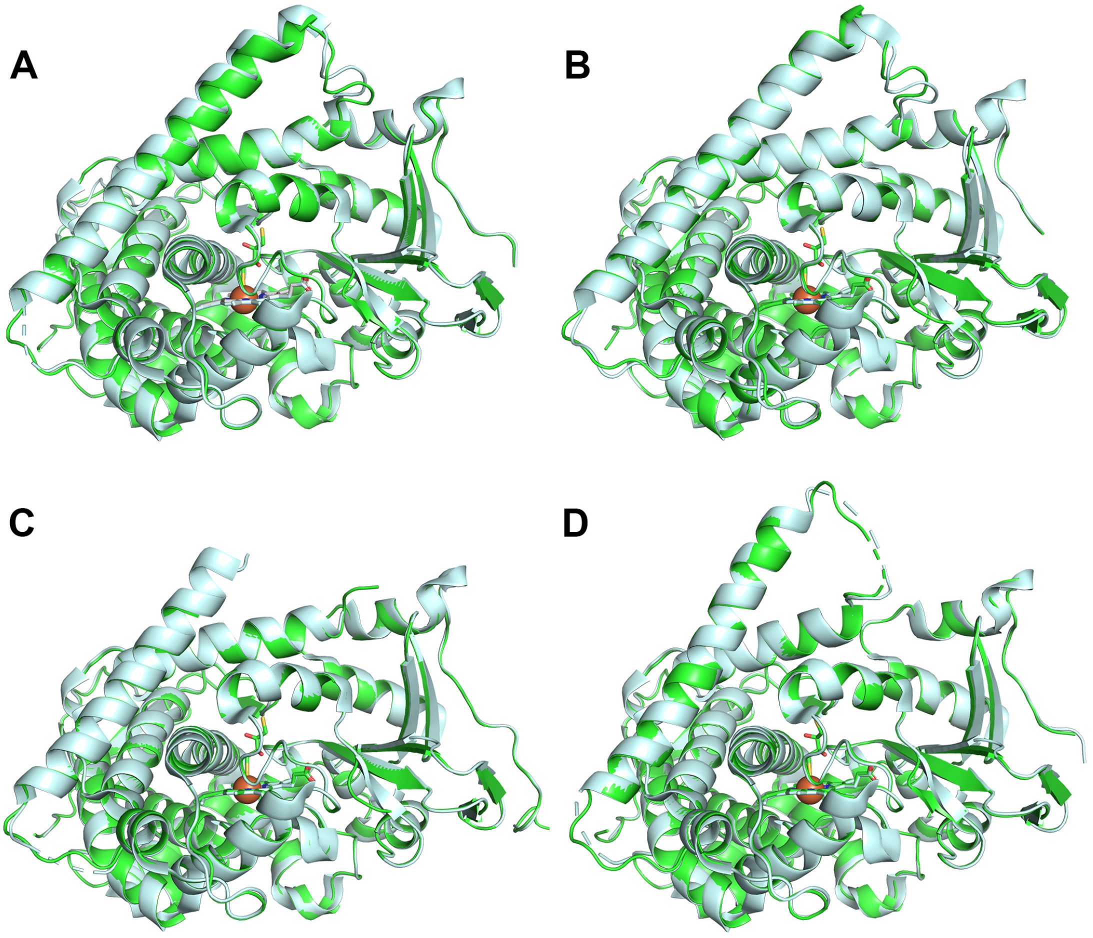


**S1 Fig. Comparison between the structure of the CYP BM3 M11 mutant in complex with DTT and the M11 structure without organic ligand (PDB entry 5E9Z).** Protein chains are shown as cartoons with the heme group and DTT in sticks. DTT-bound M11 is colored green and 5E9Z in pale cyan. Root-mean-square deviations are presented in S1 Table. (A) Chain A from both structures. (B) Chain B from both structures. (C) Chain C from both structures. (D) Chain D from both structures.
