# Supplementary material for: Structural analysis of Cytochrome P450 BM3 mutant M11 in complex with dithiothreitol
Source: PLoS One. 2019 May 24;14(5):e0217292. doi: 10.1371/journal.pone.0217292 (PMC6534296; doi:10.1371/journal.pone.0217292)
Supplement: S2 Fig — Potential H-bonds between DTT and Arg190 and three water molecules are shown as dashed black lines. Part of the protein is shown as cartoon (chain A in green and chain B in cyan), DTT and Arg190 in sticks. (PDF) [file pone.0217292.s002.pdf]

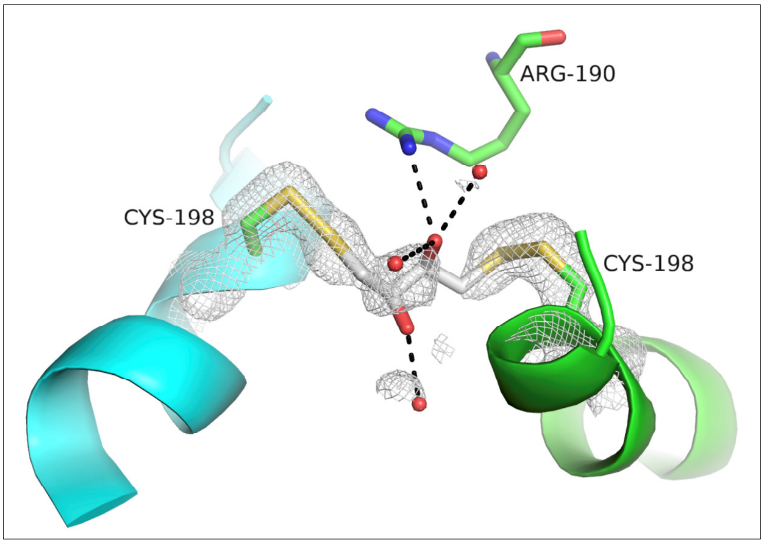

S2 Fig. The DTT molecule bridging the A and B chain in the CYP BM3 M11 structure. Potential H-bonds between DTT and Arg190 and three water molecules are shown as dashed black lines. Part of the protein is shown as cartoon (chain A in green and chain B in cyan), DTT and Arg190 in sticks.
