# Supplementary material for: Structural analysis of Cytochrome P450 BM3 mutant M11 in complex with dithiothreitol
Source: PLoS One. 2019 May 24;14(5):e0217292. doi: 10.1371/journal.pone.0217292 (PMC6534296; doi:10.1371/journal.pone.0217292)
Supplement: S3 Fig — The heme binding site with DTT is shown with 2Fo−Fc omit electron density maps contoured at 1σ and carved at 2.0 A° around DTT. Helices F, G and I are shown in cartoon representation and DTT, heme group and Cys400 in stick representation. (A) Chain A. (B) Chain B. (C) Chain C. (D) Chain D. (PDF) [file pone.0217292.s003.pdf]

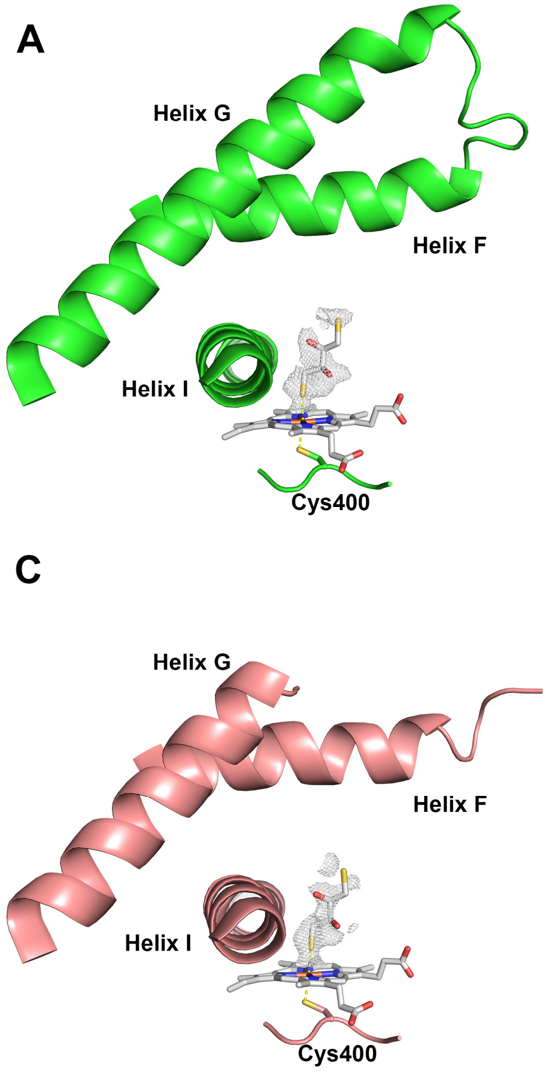

S3 Fig. Structure of CYP450 BM3 M11 in complex with DTT. The heme binding site with DTT is shown with 2Fo–Fc omit electron density maps contoured at 1σ and carved at 2.0 Å around DTT. Helices F, G and I are shown in cartoon representation and DTT, heme group and Cys400 in stick representation. (A) Chain A. (B) Chain B. (C) Chain C. (D) Chain D.
