# Supplementary material for: Structural analysis of Cytochrome P450 BM3 mutant M11 in complex with dithiothreitol
Source: PLoS One. 2019 May 24;14(5):e0217292. doi: 10.1371/journal.pone.0217292 (PMC6534296; doi:10.1371/journal.pone.0217292)
Supplement: S4 Fig — PDB entry 3I8R, chains A, B and C; and PDB entry 3I9U. (PDF) [file pone.0217292.s004.pdf]

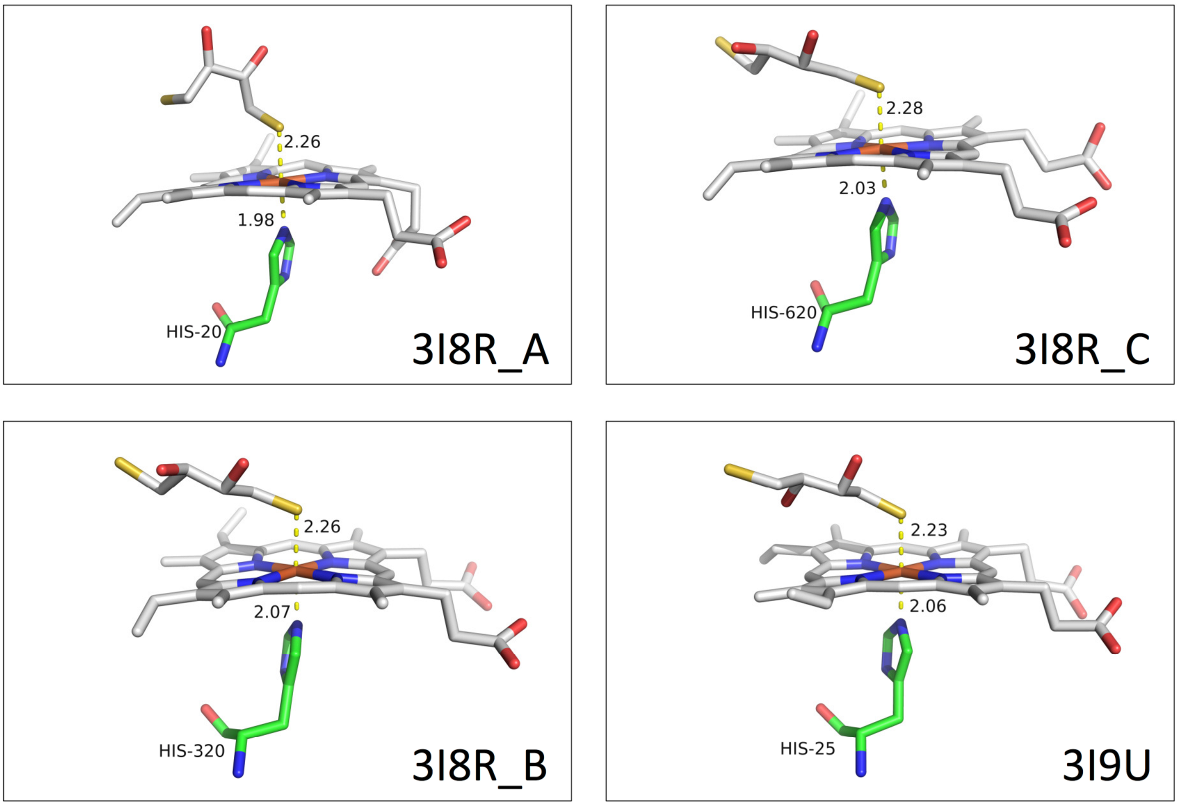

S4 Fig. Structures of mercapto-containing ligands coordinating to the Fe atom in a porphyrin group. PDB entry 3I8R, chains A, B and C; and PDB entry 3I9U.
