# Supplementary material for: Structural analysis of Cytochrome P450 BM3 mutant M11 in complex with dithiothreitol
Source: PLoS One. 2019 May 24;14(5):e0217292. doi: 10.1371/journal.pone.0217292 (PMC6534296; doi:10.1371/journal.pone.0217292)
Supplement: S5 Fig — PDB entries 4HPA, 4HPB, 4HPC, 4HPD, 4V2K, 2EVP, 2PBJ, and 2FKZ. (PDF) [file pone.0217292.s005.pdf]

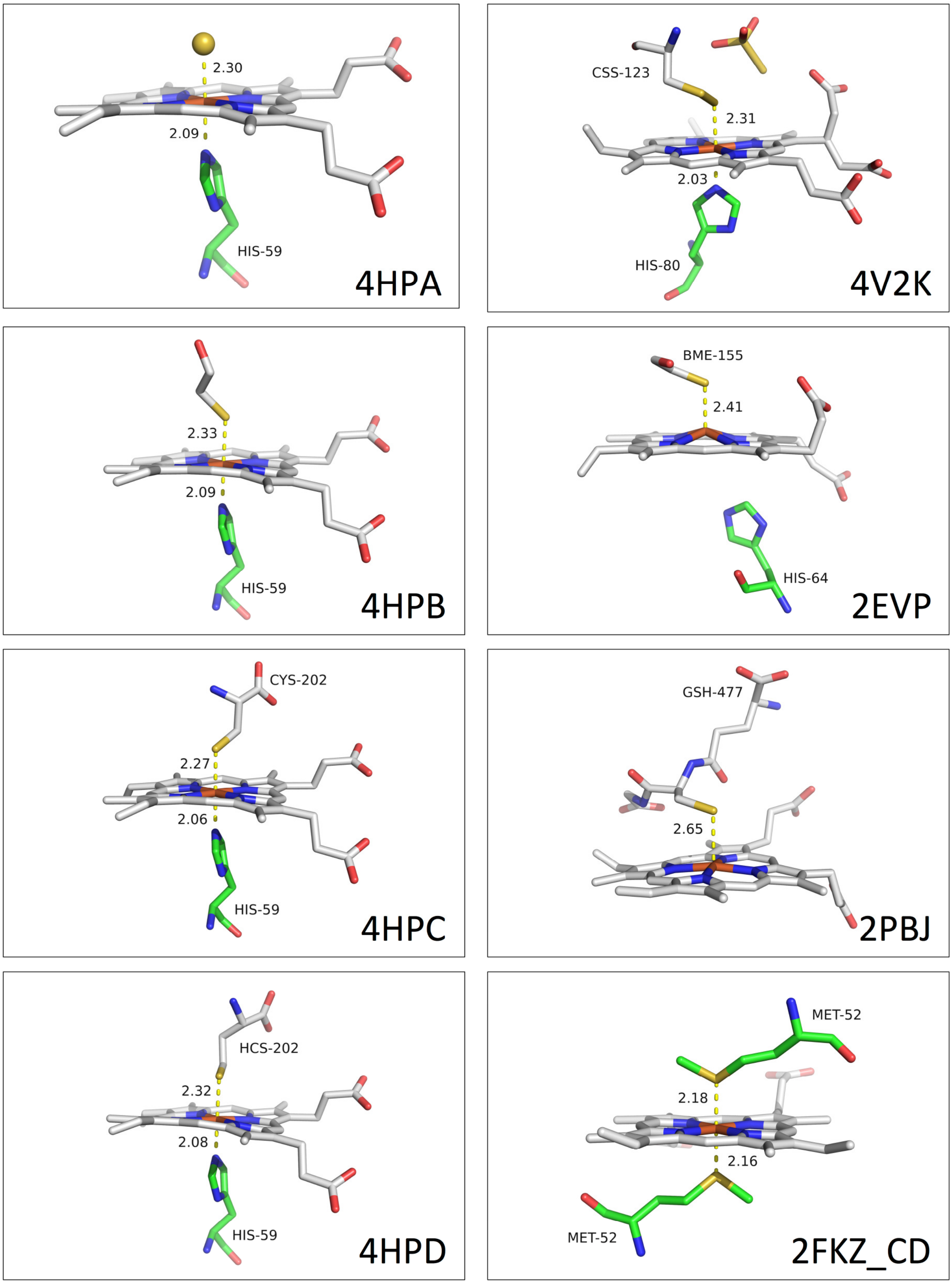

S5 Fig. Structures from the Protein Data Bank of sulfur-containing ligands coordinating to the Fe atom in a porphyrin group. PDB entries 4HPA, 4HPB, 4HPC, 4HPD, 4V2K, 2EVP, 2PBJ, and 2FKZ.
