# Supplementary material for: Structural analysis of Cytochrome P450 BM3 mutant M11 in complex with dithiothreitol
Source: PLoS One. 2019 May 24;14(5):e0217292. doi: 10.1371/journal.pone.0217292 (PMC6534296; doi:10.1371/journal.pone.0217292)
Supplement: S6 Fig — CORNAY, JELMIW, WAHLAU, and WAHLEY. (PDF) [file pone.0217292.s006.pdf]

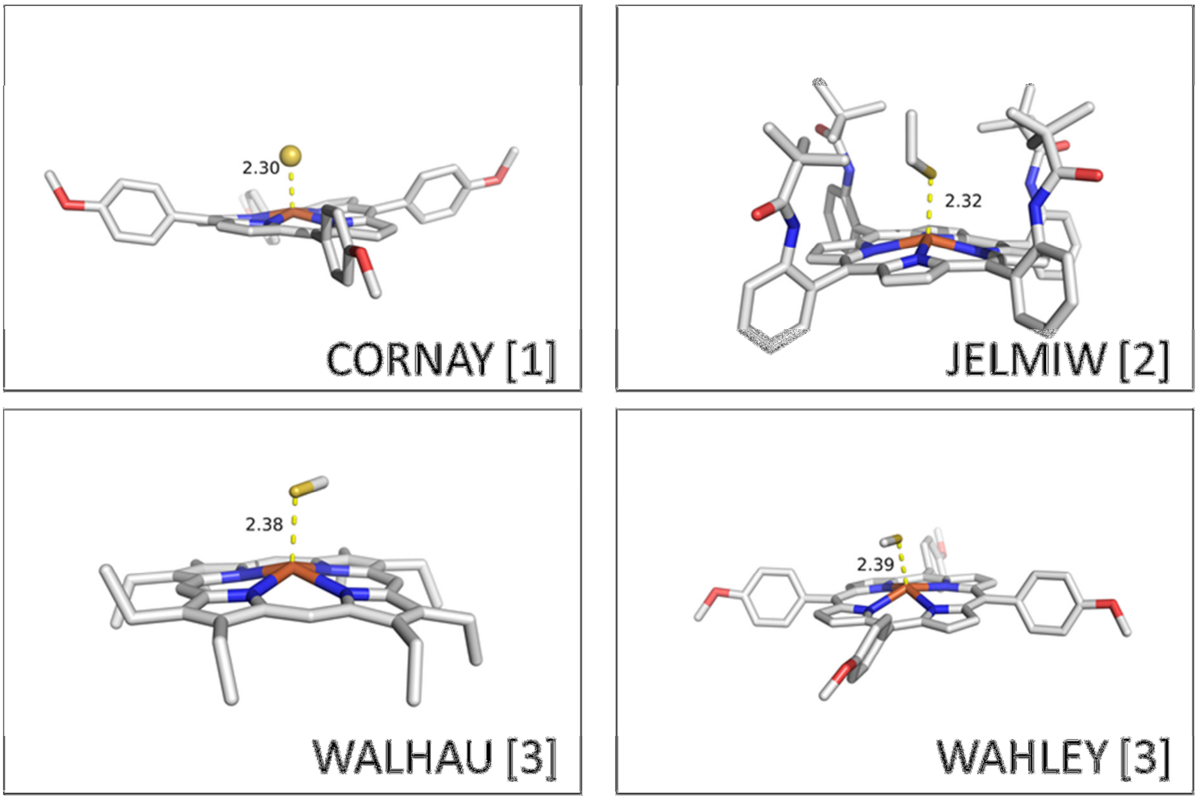

S6 Fig. Structures from the Cambridge Structural Database containing a porphyrin ring with axial sulfur-containing compounds. CORNAY, JELMIW, WAHLAU, and WAHLEY.
