# Supplementary material for: Structural analysis of Cytochrome P450 BM3 mutant M11 in complex with dithiothreitol
Source: PLoS One. 2019 May 24;14(5):e0217292. doi: 10.1371/journal.pone.0217292 (PMC6534296; doi:10.1371/journal.pone.0217292)
Supplement: S1 Table — Root-mean-square deviations (RMSDs, in Å) between chains. Alignment is performed and RMSDs are calculated for Cα atoms using Pymol (Version 2.0.6, Schrodinger). Structures are shown in S1 Fig. (PDF) [file pone.0217292.s007.pdf]

S1 Table. The present structure of CYP BM3 M11 in complex with DTT, compared to the structure without ligand (5E9Z). Root-mean-square deviations (RMSDs, in Å) between chains. Alignment is performed and RMSDs are calculated for C_α atoms using Pymol (Version 2.0.6, Schrodinger). Structures are shown in S1 Fig.

	M11(A) – 5E9Z(A)	M11(B) – 5E9Z(B)	M11(C) – 5E9Z(C)	M11(D) – 5E9Z(D)
**RMSD [Å]**	0.198	0.220	0.174	0.174
