# Supplementary material for: Structural analysis of Cytochrome P450 BM3 mutant M11 in complex with dithiothreitol
Source: PLoS One. 2019 May 24;14(5):e0217292. doi: 10.1371/journal.pone.0217292 (PMC6534296; doi:10.1371/journal.pone.0217292)
Supplement: S2 Table — Chain A was used for comparison. Alignment is performed and RMSDs are calculated for Cα atoms using Pymol (Version 2.0.6, Schrodinger). (PDF) [file pone.0217292.s008.pdf]

S2 Table. Root-mean-square deviations (RMSDs, in Å) between the four protein chains in the asymmetric unit of the present CYP BM3 M11 structure. Chain A was used for comparison. Alignment is performed and RMSDs are calculated for C_α atoms using Pymol (Version 2.0.6, Schrodinger).

**Chain A to**	**Chain B**	**Chain C**	**Chain D**
**RMSD [Å]**	0.142	0.208	0.207
