# Supplementary material for: Structural analysis of Cytochrome P450 BM3 mutant M11 in complex with dithiothreitol
Source: PLoS One. 2019 May 24;14(5):e0217292. doi: 10.1371/journal.pone.0217292 (PMC6534296; doi:10.1371/journal.pone.0217292)
Supplement: S3 Table — Literature references in S1 File. (PDF) [file pone.0217292.s009.pdf]

S3 Table. Cytochrome P450 BM3 structures from PDB. Literature references in S1 File.

1JME[4], 1P0V[5], 1P0W[5], 1P0X[5], 1SMI[6], 1SMJ[6], 1YQO[7], 1YQP[7], 2IJ2[8], 2IJ3[8], 2IJ4[8], 2J1M[9], 2J4S[9], 2UWH[10], 2X7Y[11], 2X80[11], 3CBD[12], 3DGI[13], 3EKB[14], 3EKF[14], 3HF2[15], 3KX3[16], 3KX4[16], 3KX5[16], 3M4V[17], 3NPL[18], 3PSX[19], 3QI8[20], 4DQK[21], 4DQL[21], 4DTW[22], 4DTY[22], 4DTZ[22], 4DU2[22], 4DUA[22], 4DUB[22], 4DUC[22], 4DUD[22], 4DUE[22], 4DUF[22], 4H23[23], 4H24[23], 4HGF[24], 4HGG[24], 4HGH[24], 4HGI[24], 4HGJ[24], 4KEW[25], 4KEY[25], 4KF0[25], 4KF2[25], 4O4P[26], 4RSN[27], 4ZF6[28], 4ZF8[28], 4ZFA[28], 4ZFB[28], 5DYP[29], 5DYZ[29], 5E78[30], 5E7Y[30], 5E9Z[31], 5JQ2[32], 5JQU[33], 5JQV[33], 5JTD[32], 5OG9[34], 5XA3[35], 5XHJ[36], 5ZIS[37]
