# Supplementary material for: Structural analysis of Cytochrome P450 BM3 mutant M11 in complex with dithiothreitol
Source: PLoS One. 2019 May 24;14(5):e0217292. doi: 10.1371/journal.pone.0217292 (PMC6534296; doi:10.1371/journal.pone.0217292)
Supplement: S1 File — References used in S6 Fig and S3 Table. (PDF) [file pone.0217292.s010.pdf]

**S1 File. Literature references.** References used in S6 Fig and S3 Table.


1. English DR, Hendrickson DN, Suslick KS, Eigenbrot CW, Scheidt WR. Low-spin five-coordinate ferric porphyrin complex: [5, 10, 15, 20-tetrakis(4-methoxyphenyl)porphyrinato](hydrosulfido)iron(III). J Am Chem Soc. 1984;106:7258-9.

2. Schappacher M, Ricard L, Fischer J, Weiss R, Montiel-Montoya R, Bill E, Trautwein AX. Synthesis, structure, and spectroscopic properties of five-coordinate mercaptoiron(II) porphyrins. Models for the reduced state of cytochrome P450. Inorg Chem. 1989;28:4639-45.

3. Pavlik JW, Noll BC, Oliver AG, Schulz CE, Scheidt WR. Hydrosulfide (HS−) Coordination in Iron Porphyrinates. Inorg Chem. 2010;49:1017-26.

4. Ost TW, Munro AW, Mowat CG, Taylor PR, Pesseguiero A, Fulco AJ, Cho AK, Cheesman MA, Walkinshaw MD, Chapman SK. Structural and spectroscopic analysis of the F393H mutant of flavocytochrome P450 BM3. Biochemistry. 2001;40:13430-8.

5. Ost TW, Clark J, Mowat CG, Miles CS, Walkinshaw MD, Reid GA, Chapman SK, Daff S. Oxygen activation and electron transfer in flavocytochrome P450 BM3. J Am Chem Soc. 2003;125:15010-20.

6. Joyce MG, Girvan HM, Munro AW, Leys D. A single mutation in cytochrome P450 BM3 induces the conformational rearrangement seen upon substrate binding in the wild-type enzyme. J Biol Chem. 2004;279:23287-93.

7. Clark JP, Miles CS, Mowat CG, Walkinshaw MD, Reid GA, Daff SN, Chapman SK. The role of Thr268 and Phe393 in cytochrome P450 BM3. J Inorg Biochem. 2006;100:1075-90.

8. Girvan HM, Seward HE, Toogood HS, Cheesman MR, Leys D, Munro AW. Structural and spectroscopic characterization of P450 BM3 mutants with unprecedented P450 heme iron

ligand sets. New heme ligation states influence conformational equilibria in P450 BM3. J Biol Chem. 2007;282:564-72.

9. Kuper J, Wong TS, Roccatano D, Wilmanns M, Schwaneberg U. Understanding a mechanism of organic cosolvent inactivation in heme monooxygenase P450 BM-3. J Am Chem Soc. 2007;129:5786-7.

10. Huang WC, Westlake AC, Marechal JD, Joyce MG, Moody PC, Roberts GC. Filling a hole in cytochrome P450 BM3 improves substrate binding and catalytic efficiency. J Mol Biol. 2007;373:633-51.

11. Kuper J, Tee KL, Wilmanns M, Roccatano D, Schwaneberg U, Wong TS. The role of active-site Phe87 in modulating the organic co-solvent tolerance of cytochrome P450 BM3 monooxygenase. Acta Crystallogr Sect F Struct Biol Cryst Commun. 2012;68:1013-7.

12. Fasan R, Meharenna YT, Snow CD, Poulos TL, Arnold FH. Evolutionary History of a Specialized P450 Propane Monooxygenase. J Mol Biol. 2008;383:1069-80.

13. Roberts AG, Katayama J, Kaspera R, Ledwitch KV, Le Trong I, Stenkamp RE, Thompson JA, Totah RA. The role of cytochrome P450 BM3 phenylalanine-87 and threonine-268 in binding organic hydroperoxides. Biochim Biophys Acta. 2016;1860:669-77.

14. Girvan HM, Toogood HS, Littleford RE, Seward HE, Smith WE, Ekanem IS, Leys D, Cheesman MR, Munro AW. Novel haem co-ordination variants of flavocytochrome P450BM3. Biochem J. 2009;417:65-76.

15. Whitehouse CJ, Bell SG, Yang W, Yorke JA, Blanford CF, Strong AJ, Morse EJ, Bartlam M, Rao Z, Wong LL. A highly active single-mutation variant of P450BM3 (CYP102A1). ChemBioChem. 2009;10:1654-6.

16. Girvan HM, Levy CW, Williams P, Fisher K, Cheesman MR, Rigby SE, Leys D, Munro AW. Glutamate-haem ester bond formation is disfavoured in flavocytochrome P450 BM3: characterization of glutamate substitution mutants at the haem site of P450 BM3. Biochem J. 2010;427:455-66.

17. Whitehouse CJ, Yang W, Yorke JA, Rowlatt BC, Strong AJ, Blanford CF, Bell SG, Bartlam M, Wong LL, Rao Z. Structural basis for the properties of two single-site proline mutants of CYP102A1 (P450BM3). ChemBioChem. 2010;11:2549-56.

18. Ener ME, Lee YT, Winkler JR, Gray HB, Cheruzel L. Photooxidation of cytochrome P450-BM3. Proc Natl Acad Sci USA. 2010;107:18783-6.

19. Whitehouse CJ, Yang W, Yorke JA, Tufton HG, Ogilvie LC, Bell SG, Zhou W, Bartlam M, Rao Z, Wong LL. Structure, electronic properties and catalytic behaviour of an activity-enhancing CYP102A1 (P450(BM3)) variant. Dalton Trans. 2011;40:10383-96.

20. Rentmeister A, Brown TR, Snow CD, Carbone MN, Arnold FH. Engineered Bacterial Mimics of Human Drug Metabolizing Enzyme CYP2C9. ChemCatChem. 2011;3:1065-71.

21. Joyce MG, Ekanem IS, Roitel O, Dunford AJ, Neeli R, Girvan HM, Baker GJ, Curtis RA, Munro AW, Leys D. The crystal structure of the FAD/NADPH-binding domain of flavocytochrome P450 BM3. FEBS J. 2012;279:1694-706.

22. Brustad EM, Lelyveld VS, Snow CD, Crook N, Jung ST, Martinez FM, Scholl TJ, Jasanoff A, Arnold FH. Structure-guided directed evolution of highly selective p450-based magnetic resonance imaging sensors for dopamine and serotonin. J Mol Biol. 2012;422:245-62.

23. Coelho PS, Wang ZJ, Ener ME, Baril SA, Kannan A, Arnold FH, Brustad EM. A serine-substituted P450 catalyzes highly efficient carbene transfer to olefins in vivo. Nat Chem Biol. 2013;9:485-7.

24. Shehzad A, Panneerselvam S, Linow M, Bocola M, Roccatano D, Mueller-Dieckmann J, Wilmanns M, Schwaneberg U. P450 BM3 crystal structures reveal the role of the charged surface residue Lys/Arg184 in inversion of enantioselective styrene epoxidation. Chem Commun. 2013;49:4694-6.

25. Butler CF, Peet C, Mason AE, Voice MW, Leys D, Munro AW. Key mutations alter the cytochrome P450 BM3 conformational landscape and remove inherent substrate bias. J Biol Chem. 2013;288:25387-99.

26. Butler CF, Peet C, McLean KJ, Baynham MT, Blankley RT, Fisher K, Rigby SE, Leys D, Voice MW, Munro AW. Human P450-like oxidation of diverse proton pump inhibitor drugs by 'gatekeeper' mutants of flavocytochrome P450 BM3. Biochem J. 2014;460:247-59.

27. Ren X, Yorke JA, Taylor E, Zhang T, Zhou W, Wong LL. Drug Oxidation by Cytochrome P450BM3 : Metabolite Synthesis and Discovering New P450 Reaction Types. Chemistry. 2015;21:15039-47.

28. Geronimo I, Denning CA, Rogers WE, Othman T, Huxford T, Heidary DK, Glazer EC, Payne CM. Effect of Mutation and Substrate Binding on the Stability of Cytochrome P450BM3 Variants. Biochemistry. 2016;55:3594-606.

29. Di Nardo G, Dell'Angelo V, Catucci G, Sadeghi SJ, Gilardi G. Subtle structural changes in the Asp251Gly/Gln307His P450 BM3 mutant responsible for new activity toward diclofenac, tolbutamide and ibuprofen. Arch Biochem Biophys. 2016;602:106-15.

30. Panneerselvam S, Shehzad A, Mueller-Dieckmann J, Wilmanns M, Bocola M, Davari MD, Schwaneberg U. Crystallographic insights into a cobalt (III) sepulchrate based alternative cofactor system of P450 BM3 monooxygenase. Biochim Biophys Acta. 2018;1866:134-40.

31. Capoferri L, Leth R, ter Haar E, Mohanty AK, Grootenhuis PD, Vottero E, Commandeur JN, Vermeulen NP, Jorgensen FS, Olsen L, Geerke DP. Insights into regioselective metabolism of mefenamic acid by cytochrome P450 BM3 mutants through crystallography, docking, molecular dynamics, and free energy calculations. Proteins. 2016;84:383-96.

32. Spradlin J, Lee D, Mahadevan S, Mahomed M, Tang L, Lam Q, Colbert A, Shafaat OS, Goodin D, Kloos M, Kato M, Cheruzel LE. Insights into an efficient light-driven hybrid P450 BM3 enzyme from crystallographic, spectroscopic and biochemical studies. Biochim Biophys Acta. 2016;1864:1732-8.

33. Reynolds EW, McHenry MW, Cannac F, Gober JG, Snow CD, Brustad EM. An Evolved Orthogonal Enzyme/Cofactor Pair. J Am Chem Soc. 2016;138:12451-8.

34. Acevedo-Rocha CG, Gamble CG, Lonsdale R, Li A, Nett N, Hoebenreich S, Lingnau JB, Wirtz C, Fares C, Hinrichs H, Deege A, Mulholland AJ, Nov Y, Leys D, McLean KJ, Munro AW, Reetz MT. P450-Catalyzed Regio- and Diastereoselective Steroid Hydroxylation: Efficient Directed Evolution Enabled by Mutability Landscaping. ACS Catalysis. 2018;8:3395-410.

35. Shoji O, Yanagisawa S, Stanfield JK, Suzuki K, Cong Z, Sugimoto H, Shiro Y, Watanabe Y. Direct Hydroxylation of Benzene to Phenol by Cytochrome P450BM3 Triggered by Amino Acid Derivatives. Angew Chem Int Ed. 2017;56:10324-9.

36. Suzuki K, Stanfield JK, Shoji O, Yanagisawa S, Sugimoto H, Shiro Y, Watanabe Y. Control of stereoselectivity of benzylic hydroxylation catalysed by wild-type cytochrome P450BM3 using decoy molecules. Catalysis Science & Technology 2017;7:3332-8.

37. Omura K, Aiba Y, Onoda H, Stanfield JK, Ariyasu S, Sugimoto H, Shiro Y, Shoji O, Watanabe Y. Reconstitution of full-length P450BM3 with an artificial metal complex by utilising the transpeptidase Sortase A. Chem Commun. 2018;54:7892-5.
