# Supplementary material for: Structural analysis of Cytochrome P450 BM3 mutant M11 in complex with dithiothreitol
Source: PLoS One. 2019 May 24;14(5):e0217292. doi: 10.1371/journal.pone.0217292 (PMC6534296; doi:10.1371/journal.pone.0217292)
Supplement: S2 File — Copy of the PDB X-ray Structure Validation Report. (PDF) [file pone.0217292.s011.pdf]

Full wwPDB X-ray Structure Validation Report (i)

Nov 28, 2018 – 09:43 AM GMT

PDB ID	:	6IAO
Title	:	Structure of Cytochrome P450 BM3 M11 mutant in complex with DTT at resolution 2.16A
Deposited on	:	2018-11-27
Resolution	:	2.16 Å(reported)

This is a Full wwPDB X-ray Structure Validation Report.

This report is produced by the wwPDB biocuration pipeline after annotation of the structure.

We welcome your comments at *validation@mail.wwpdb.org*
A user guide is available at
https://www.wwpdb.org/validation/2017/XrayValidationReportHelp
with specific help available everywhere you see the (i) symbol.

CONFIDENTIAL VALIDATION REPORT

The following versions of software and data (see references (i)) were used in the production of this report:

MolProbity	:	4.02b-467
Mogul	:	1.7.3 (157068), CSD as539be (2018)
Xtriage (Phenix)	:	1.13
EDS	:	rb-20031633
Percentile statistics	:	20171227.v01 (using entries in the PDB archive December 27th 2017)
Refmac	:	5.8.0158
CCP4	:	7.0 (Gargrove)
Ideal geometry (proteins)	:	Engh & Huber (2001)
Ideal geometry (DNA, RNA)	:	Parkinson et al. (1996)
Validation Pipeline (wwPDB-VP)	:	rb-20031633

1 Overall quality at a glance ⓘ

The following experimental techniques were used to determine the structure:
X-RAY DIFFRACTION

The reported resolution of this entry is 2.16 Å.

Percentile scores (ranging between 0-100) for global validation metrics of the entry are shown in the following graphic. The table shows the number of entries on which the scores are based.

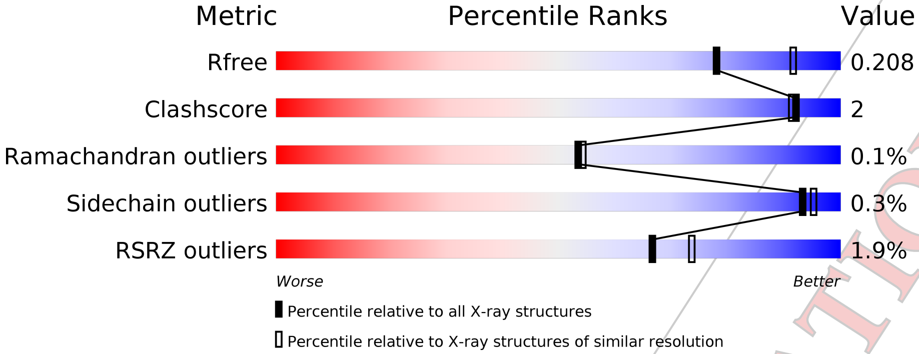

Metric	Whole archive (#Entries)	Similar resolution (#Entries, resolution range(Å))
R_{free}	111664	1287 (2.16-2.16)
Clashscore	122126	1390 (2.16-2.16)
Ramachandran outliers	120053	1368 (2.16-2.16)
Sidechain outliers	120020	1367 (2.16-2.16)
RSRZ outliers	108989	1262 (2.16-2.16)

The table below summarises the geometric issues observed across the polymeric chains and their fit to the electron density. The red, orange, yellow and green segments on the lower bar indicate the fraction of residues that contain outliers for >=3, 2, 1 and 0 types of geometric quality criteria. A grey segment represents the fraction of residues that are not modelled. The numeric value for each fraction is indicated below the corresponding segment, with a dot representing fractions <=5% The upper red bar (where present) indicates the fraction of residues that have poor fit to the electron density. The numeric value is given above the bar.

Mol	Chain	Length	Quality of chain
1	A	507	% 86% • 10%
1	B	507	% 87% • 11%
1	C	507	4% 84% 5% 11%
1	D	507	% 84% • 11%

CONFIDENTIAL VALIDATION REPORT

2 Entry composition (i)

There are 7 unique types of molecules in this entry. The entry contains 16073 atoms, of which 0 are hydrogens and 0 are deuteriums.

In the tables below, the ZeroOcc column contains the number of atoms modelled with zero occupancy, the AltConf column contains the number of residues with at least one atom in alternate conformation and the Trace column contains the number of residues modelled with at most 2 atoms.

- Molecule 1 is a protein called Bifunctional cytochrome P450/NADPH–P450 reductase.

Mol	Chain	Residues	Atoms					ZeroOcc	AltConf	Trace
1	A	455	Total 3670	C 2346	N 625	O 680	S 19	0	5	0
1	B	452	Total 3653	C 2332	N 624	O 679	S 18	0	5	0
1	C	450	Total 3625	C 2319	N 617	O 669	S 20	0	5	0
1	D	450	Total 3599	C 2300	N 608	O 673	S 18	0	0	0

There are 176 discrepancies between the modelled and reference sequences:

Chain	Residue	Modelled	Actual	Comment	Reference
A	-34	MET	-	initiating methionine	UNP P14779
A	-33	GLY	-	expression tag	UNP P14779
A	-32	SER	-	expression tag	UNP P14779
A	-31	SER	-	expression tag	UNP P14779
A	-30	HIS	-	expression tag	UNP P14779
A	-29	HIS	-	expression tag	UNP P14779
A	-28	HIS	-	expression tag	UNP P14779
A	-27	HIS	-	expression tag	UNP P14779
A	-26	HIS	-	expression tag	UNP P14779
A	-25	HIS	-	expression tag	UNP P14779
A	-24	SER	-	expression tag	UNP P14779
A	-23	SER	-	expression tag	UNP P14779
A	-22	GLY	-	expression tag	UNP P14779
A	-21	LEU	-	expression tag	UNP P14779
A	-20	VAL	-	expression tag	UNP P14779
A	-19	PRO	-	expression tag	UNP P14779
A	-18	ARG	-	expression tag	UNP P14779
A	-17	GLY	-	expression tag	UNP P14779
A	-16	SER	-	expression tag	UNP P14779
A	-15	HIS	-	expression tag	UNP P14779
A	-14	MET	-	expression tag	UNP P14779

Continued on next page...

CONFIDENTIAL VALIDATION REPORT

Continued from previous page...

Chain	Residue	Modelled	Actual	Comment	Reference
A	-13	ALA	-	expression tag	UNP P14779
A	-12	SER	-	expression tag	UNP P14779
A	-11	MET	-	expression tag	UNP P14779
A	-10	THR	-	expression tag	UNP P14779
A	-9	GLY	-	expression tag	UNP P14779
A	-8	GLY	-	expression tag	UNP P14779
A	-7	GLN	-	expression tag	UNP P14779
A	-6	GLN	-	expression tag	UNP P14779
A	-5	MET	-	expression tag	UNP P14779
A	-4	GLY	-	expression tag	UNP P14779
A	-3	ARG	-	expression tag	UNP P14779
A	-2	GLY	-	expression tag	UNP P14779
A	-1	SER	-	expression tag	UNP P14779
A	47	LEU	ARG	conflict	UNP P14779
A	64	GLY	GLU	conflict	UNP P14779
A	81	ILE	PHE	conflict	UNP P14779
A	87	VAL	PHE	conflict	UNP P14779
A	143	GLY	GLU	conflict	UNP P14779
A	188	GLN	LEU	conflict	UNP P14779
A	198	CYS	TYR	conflict	UNP P14779
A	267	VAL	GLU	conflict	UNP P14779
A	285	TYR	HIS	conflict	UNP P14779
A	415	SER	GLY	conflict	UNP P14779
B	-34	MET	-	initiating methionine	UNP P14779
B	-33	GLY	-	expression tag	UNP P14779
B	-32	SER	-	expression tag	UNP P14779
B	-31	SER	-	expression tag	UNP P14779
B	-30	HIS	-	expression tag	UNP P14779
B	-29	HIS	-	expression tag	UNP P14779
B	-28	HIS	-	expression tag	UNP P14779
B	-27	HIS	-	expression tag	UNP P14779
B	-26	HIS	-	expression tag	UNP P14779
B	-25	HIS	-	expression tag	UNP P14779
B	-24	SER	-	expression tag	UNP P14779
B	-23	SER	-	expression tag	UNP P14779
B	-22	GLY	-	expression tag	UNP P14779
B	-21	LEU	-	expression tag	UNP P14779
B	-20	VAL	-	expression tag	UNP P14779
B	-19	PRO	-	expression tag	UNP P14779
B	-18	ARG	-	expression tag	UNP P14779
B	-17	GLY	-	expression tag	UNP P14779
B	-16	SER	-	expression tag	UNP P14779

Continued on next page...

CONFIDENTIAL VALIDATION REPORT

Continued from previous page...

Chain	Residue	Modelled	Actual	Comment	Reference
B	-15	HIS	-	expression tag	UNP P14779
B	-14	MET	-	expression tag	UNP P14779
B	-13	ALA	-	expression tag	UNP P14779
B	-12	SER	-	expression tag	UNP P14779
B	-11	MET	-	expression tag	UNP P14779
B	-10	THR	-	expression tag	UNP P14779
B	-9	GLY	-	expression tag	UNP P14779
B	-8	GLY	-	expression tag	UNP P14779
B	-7	GLN	-	expression tag	UNP P14779
B	-6	GLN	-	expression tag	UNP P14779
B	-5	MET	-	expression tag	UNP P14779
B	-4	GLY	-	expression tag	UNP P14779
B	-3	ARG	-	expression tag	UNP P14779
B	-2	GLY	-	expression tag	UNP P14779
B	-1	SER	-	expression tag	UNP P14779
B	47	LEU	ARG	conflict	UNP P14779
B	64	GLY	GLU	conflict	UNP P14779
B	81	ILE	PHE	conflict	UNP P14779
B	87	VAL	PHE	conflict	UNP P14779
B	143	GLY	GLU	conflict	UNP P14779
B	188	GLN	LEU	conflict	UNP P14779
B	198	CYS	TYR	conflict	UNP P14779
B	267	VAL	GLU	conflict	UNP P14779
B	285	TYR	HIS	conflict	UNP P14779
B	415	SER	GLY	conflict	UNP P14779
C	-34	MET	-	initiating methionine	UNP P14779
C	-33	GLY	-	expression tag	UNP P14779
C	-32	SER	-	expression tag	UNP P14779
C	-31	SER	-	expression tag	UNP P14779
C	-30	HIS	-	expression tag	UNP P14779
C	-29	HIS	-	expression tag	UNP P14779
C	-28	HIS	-	expression tag	UNP P14779
C	-27	HIS	-	expression tag	UNP P14779
C	-26	HIS	-	expression tag	UNP P14779
C	-25	HIS	-	expression tag	UNP P14779
C	-24	SER	-	expression tag	UNP P14779
C	-23	SER	-	expression tag	UNP P14779
C	-22	GLY	-	expression tag	UNP P14779
C	-21	LEU	-	expression tag	UNP P14779
C	-20	VAL	-	expression tag	UNP P14779
C	-19	PRO	-	expression tag	UNP P14779
C	-18	ARG	-	expression tag	UNP P14779

Continued on next page...

CONFIDENTIAL VALIDATION REPORT

Continued from previous page...

Chain	Residue	Modelled	Actual	Comment	Reference
C	-17	GLY	-	expression tag	UNP P14779
C	-16	SER	-	expression tag	UNP P14779
C	-15	HIS	-	expression tag	UNP P14779
C	-14	MET	-	expression tag	UNP P14779
C	-13	ALA	-	expression tag	UNP P14779
C	-12	SER	-	expression tag	UNP P14779
C	-11	MET	-	expression tag	UNP P14779
C	-10	THR	-	expression tag	UNP P14779
C	-9	GLY	-	expression tag	UNP P14779
C	-8	GLY	-	expression tag	UNP P14779
C	-7	GLN	-	expression tag	UNP P14779
C	-6	GLN	-	expression tag	UNP P14779
C	-5	MET	-	expression tag	UNP P14779
C	-4	GLY	-	expression tag	UNP P14779
C	-3	ARG	-	expression tag	UNP P14779
C	-2	GLY	-	expression tag	UNP P14779
C	-1	SER	-	expression tag	UNP P14779
C	47	LEU	ARG	conflict	UNP P14779
C	64	GLY	GLU	conflict	UNP P14779
C	81	ILE	PHE	conflict	UNP P14779
C	87	VAL	PHE	conflict	UNP P14779
C	143	GLY	GLU	conflict	UNP P14779
C	188	GLN	LEU	conflict	UNP P14779
C	198	CYS	TYR	conflict	UNP P14779
C	267	VAL	GLU	conflict	UNP P14779
C	285	TYR	HIS	conflict	UNP P14779
C	415	SER	GLY	conflict	UNP P14779
D	-34	MET	-	initiating methionine	UNP P14779
D	-33	GLY	-	expression tag	UNP P14779
D	-32	SER	-	expression tag	UNP P14779
D	-31	SER	-	expression tag	UNP P14779
D	-30	HIS	-	expression tag	UNP P14779
D	-29	HIS	-	expression tag	UNP P14779
D	-28	HIS	-	expression tag	UNP P14779
D	-27	HIS	-	expression tag	UNP P14779
D	-26	HIS	-	expression tag	UNP P14779
D	-25	HIS	-	expression tag	UNP P14779
D	-24	SER	-	expression tag	UNP P14779
D	-23	SER	-	expression tag	UNP P14779
D	-22	GLY	-	expression tag	UNP P14779
D	-21	LEU	-	expression tag	UNP P14779
D	-20	VAL	-	expression tag	UNP P14779

Continued on next page...

CONFIDENTIAL VALIDATION REPORT

Continued from previous page...

Chain	Residue	Modelled	Actual	Comment	Reference
D	-19	PRO	-	expression tag	UNP P14779
D	-18	ARG	-	expression tag	UNP P14779
D	-17	GLY	-	expression tag	UNP P14779
D	-16	SER	-	expression tag	UNP P14779
D	-15	HIS	-	expression tag	UNP P14779
D	-14	MET	-	expression tag	UNP P14779
D	-13	ALA	-	expression tag	UNP P14779
D	-12	SER	-	expression tag	UNP P14779
D	-11	MET	-	expression tag	UNP P14779
D	-10	THR	-	expression tag	UNP P14779
D	-9	GLY	-	expression tag	UNP P14779
D	-8	GLY	-	expression tag	UNP P14779
D	-7	GLN	-	expression tag	UNP P14779
D	-6	GLN	-	expression tag	UNP P14779
D	-5	MET	-	expression tag	UNP P14779
D	-4	GLY	-	expression tag	UNP P14779
D	-3	ARG	-	expression tag	UNP P14779
D	-2	GLY	-	expression tag	UNP P14779
D	-1	SER	-	expression tag	UNP P14779
D	47	LEU	ARG	conflict	UNP P14779
D	64	GLY	GLU	conflict	UNP P14779
D	81	ILE	PHE	conflict	UNP P14779
D	87	VAL	PHE	conflict	UNP P14779
D	143	GLY	GLU	conflict	UNP P14779
D	188	GLN	LEU	conflict	UNP P14779
D	198	CYS	TYR	conflict	UNP P14779
D	267	VAL	GLU	conflict	UNP P14779
D	285	TYR	HIS	conflict	UNP P14779
D	415	SER	GLY	conflict	UNP P14779

- Molecule 2 is PROTOPORPHYRIN IX CONTAINING FE (three-letter code: HEM) (formula: $C_{34}H_{32}FeN_4O_4$).

CONFIDENTIAL VALIDATION REPORT

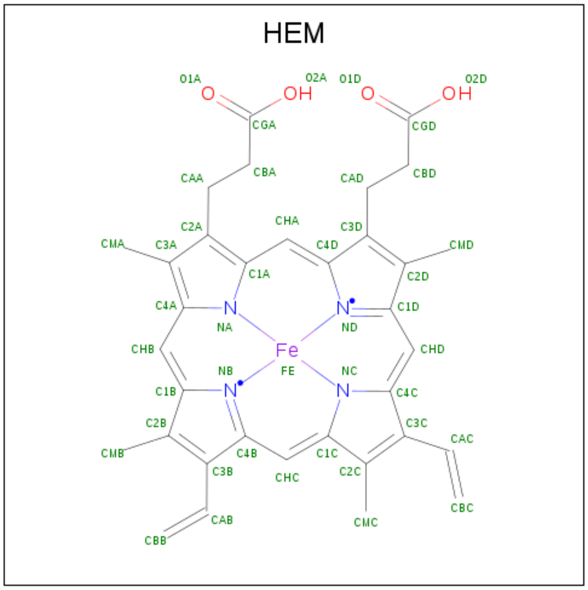

Mol	Chain	Residues	Atoms	ZeroOcc	AltConf
2	A	1	Total C Fe N O<br>43 34 1 4 4	0	0
2	B	1	Total C Fe N O<br>43 34 1 4 4	0	0
2	C	1	Total C Fe N O<br>43 34 1 4 4	0	0
2	D	1	Total C Fe N O<br>43 34 1 4 4	0	0

- Molecule 3 is 2,3-DIHYDROXY-1,4-DITHIOBUTANE (three-letter code: DTT) (formula: $C_4H_{10}O_2S_2$).

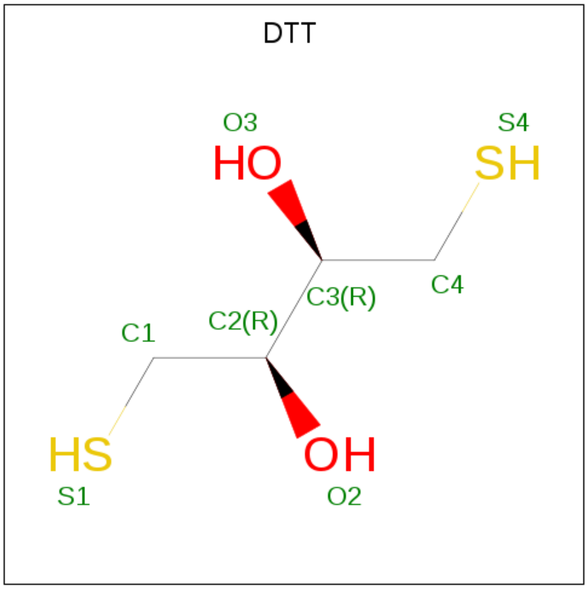

Mol	Chain	Residues	Atoms	ZeroOcc	AltConf
3	A	1	Total C O S<br>8 4 2 2	0	0
3	A	1	Total C O S<br>8 4 2 2	0	0
3	B	1	Total C O S<br>8 4 2 2	0	0
3	C	1	Total C O S<br>8 4 2 2	0	0
3	D	1	Total C O S<br>8 4 2 2	0	0

- Molecule 4 is CHLORIDE ION (three-letter code: CL) (formula: Cl).

Mol	Chain	Residues	Atoms	ZeroOcc	AltConf
4	B	5	Total Cl<br>5 5	0	0
4	A	6	Total Cl<br>6 6	0	0
4	D	5	Total Cl<br>5 5	0	0
4	C	4	Total Cl<br>4 4	0	0

- Molecule 5 is GLYCEROL (three-letter code: GOL) (formula: $C_3H_8O_3$).

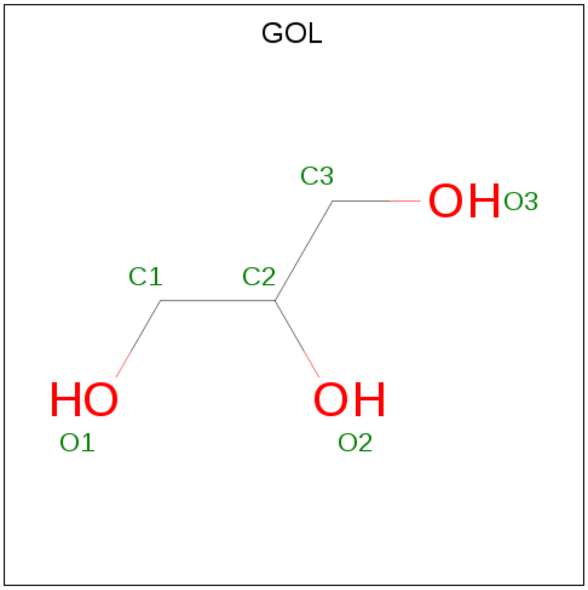

Mol	Chain	Residues	Atoms	ZeroOcc	AltConf
5	A	1	Total C O<br>6 3 3	0	0

Continued on next page...

Continued from previous page...

Mol	Chain	Residues	Atoms	ZeroOcc	AltConf
5	A	1	Total C O<br>6 3 3	0	0
5	B	1	Total C O<br>6 3 3	0	0
5	C	1	Total C O<br>6 3 3	0	0

- Molecule 6 is DI(HYDROXYETHYL)ETHER (three-letter code: PEG) (formula: $C_4H_{10}O_3$).

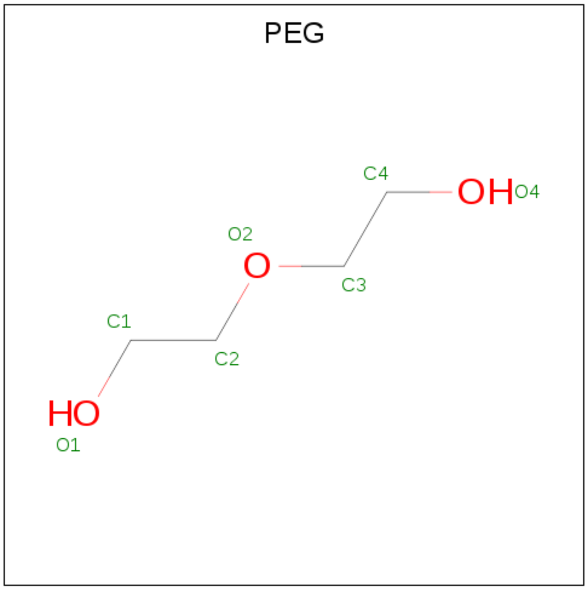

Mol	Chain	Residues	Atoms	ZeroOcc	AltConf
6	A	1	Total C O<br>7 4 3	0	0
6	C	1	Total C O<br>7 4 3	0	0

- Molecule 7 is water.

Mol	Chain	Residues	Atoms	ZeroOcc	AltConf
7	A	392	Total O<br>394 394	0	2
7	B	344	Total O<br>350 350	0	6
7	C	241	Total O<br>243 243	0	2
7	D	268	Total O<br>269 269	0	1

CONFIDENTIAL VALIDATION REPORT

3 Residue-property plots

These plots are drawn for all protein, RNA and DNA chains in the entry. The first graphic for a chain summarises the proportions of the various outlier classes displayed in the second graphic. The second graphic shows the sequence view annotated by issues in geometryand electron density. Residues are color-coded according to the number of geometric quality criteria for which they contain at least one outlier: green = 0, yellow = 1, orange = 2 and red = 3 or more. A red dot above a residue indicates a poor fit to the electron density (RSRZ > 2). Stretches of 2 or more consecutive residues without any outlier are shown as a green connector. Residues present in the sample, but not in the model, are shown in grey.

• Molecule 1: Bifunctional cytochrome P450/NADPH–P450 reductase

Chain A:

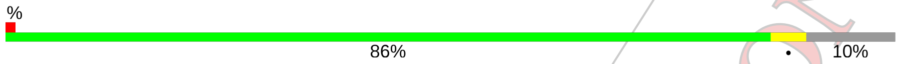

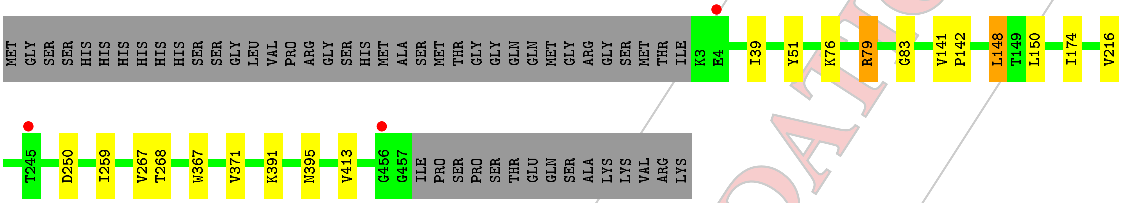

• Molecule 1: Bifunctional cytochrome P450/NADPH–P450 reductase

Chain B:

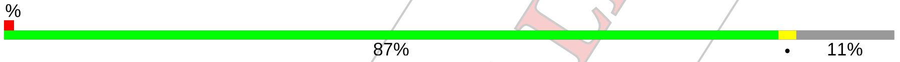

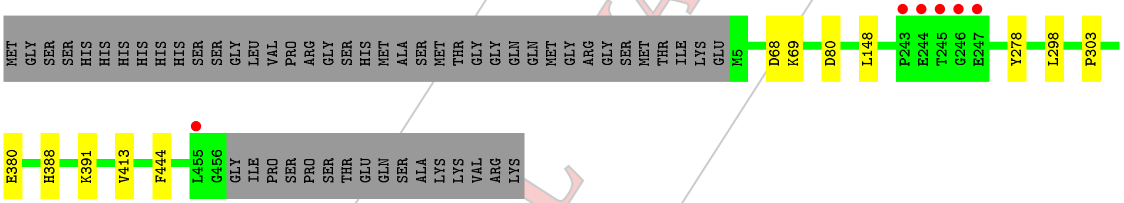

• Molecule 1: Bifunctional cytochrome P450/NADPH–P450 reductase

Chain C:

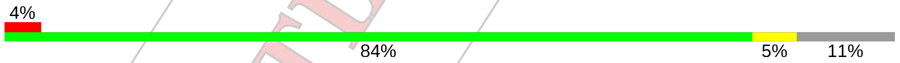

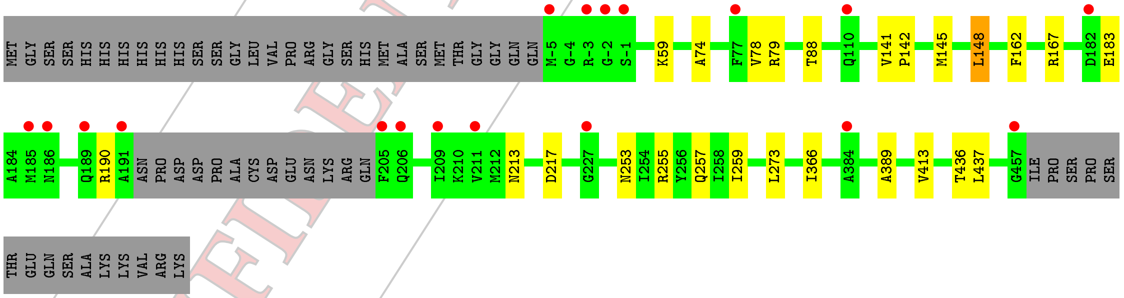

• Molecule 1: Bifunctional cytochrome P450/NADPH–P450 reductase

Chain D:

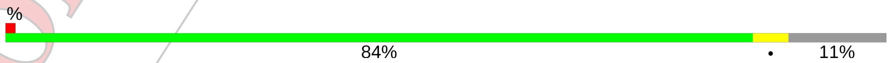

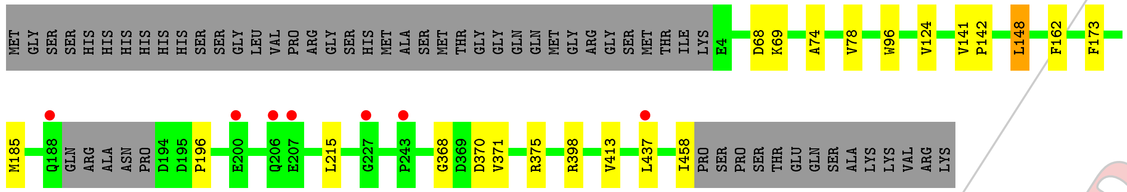

CONFIDENTIAL VALIDATION REPORT

4 Data and refinement statistics (i)

Property	Value	Source
Space group	C 1 2 1	Depositor
Cell constants<br>a, b, c, α, β, γ	377.93Å 59.94Å 95.46Å<br>90.00° 95.74° 90.00°	Depositor
Resolution (Å)	47.18 – 2.16<br>47.89 – 2.16	Depositor<br>EDS
% Data completeness<br>(in resolution range)	99.6 (47.18-2.16)<br>99.6 (47.89-2.16)	Depositor<br>EDS
R_{merge}	(Not available)	Depositor
R_{sym}	0.14	Depositor
$< I/\sigma(I) >$ [1]	1.85 (at 2.16Å)	Xtriage
Refinement program	PHENIX	Depositor
R, R_{free}	0.159 , 0.202<br>0.167 , 0.208	Depositor<br>DCC
R_{free} test set	5744 reflections (5.02%)	wwPDB-VP
Wilson B-factor (Å^2)	20.1	Xtriage
Anisotropy	0.434	Xtriage
Bulk solvent k_{sol}(e/Å^3), B_{sol}(Å^2)	0.34 , 51.4	EDS
L-test for twinning[2]	$< \|L\| >$ = 0.48, $< L^2 >$ = 0.32	Xtriage
Estimated twinning fraction	No twinning to report.	Xtriage
F_o,F_c correlation	0.95	EDS
Total number of atoms	16073	wwPDB-VP
Average B, all atoms (Å^2)	26.0	wwPDB-VP

Xtriage's analysis on translational NCS is as follows: *The largest off-origin peak in the Patterson function is 3.04% of the height of the origin peak. No significant pseudotranslation is detected.*

[1] Intensities estimated from amplitudes.

[2] Theoretical values of $< |L| >$, $< L^2 >$ for acentric reflections are 0.5, 0.333 respectively for untwinned datasets, and 0.375, 0.2 for perfectly twinned datasets.

CONFIDENTIAL VALIDATION REPORT

5 Model quality (i)

5.1 Standard geometry (i)

Bond lengths and bond angles in the following residue types are not validated in this section: GOL, PEG, DTT, HEM, CL

The Z score for a bond length (or angle) is the number of standard deviations the observed value is removed from the expected value. A bond length (or angle) with $|Z| > 5$ is considered an outlier worth inspection. RMSZ is the root-mean-square of all Z scores of the bond lengths (or angles).

Mol	Chain	Bond lengths		Bond angles	
		RMSZ	$\#\|Z\| > 5$	RMSZ	$\#\|Z\| > 5$
1	A	0.31	0/3767	0.50	0/5094
1	B	0.31	0/3747	0.49	0/5067
1	C	0.30	0/3719	0.49	0/5021
1	D	0.30	0/3679	0.48	0/4974
All	All	0.30	0/14912	0.49	0/20156

There are no bond length outliers.

There are no bond angle outliers.

There are no chirality outliers.

There are no planarity outliers.

5.2 Too-close contacts (i)

In the following table, the Non-H and H(model) columns list the number of non-hydrogen atoms and hydrogen atoms in the chain respectively. The H(added) column lists the number of hydrogen atoms added and optimized by MolProbity. The Clashes column lists the number of clashes within the asymmetric unit, whereas Symm-Clashes lists symmetry related clashes.

Mol	Chain	Non-H	H(model)	H(added)	Clashes	Symm-Clashes
1	A	3670	0	3678	12	0
1	B	3653	0	3649	6	0
1	C	3625	0	3645	13	0
1	D	3599	0	3582	13	0
2	A	43	0	30	1	0
2	B	43	0	30	1	0
2	C	43	0	30	2	0
2	D	43	0	30	1	0
3	A	16	0	17	0	0

Continued on next page...

CONFIDENTIAL VALIDATION REPORT

Continued from previous page...

Mol	Chain	Non-H	H(model)	H(added)	Clashes	Symm-Clashes
3	B	8	0	9	0	0
3	C	8	0	9	0	0
3	D	8	0	9	0	0
4	A	6	0	0	0	0
4	B	5	0	0	0	0
4	C	4	0	0	0	0
4	D	5	0	0	0	0
5	A	12	0	16	1	0
5	B	6	0	8	0	0
5	C	6	0	8	0	0
6	A	7	0	10	0	0
6	C	7	0	10	0	0
7	A	394	0	0	1	0
7	B	350	0	0	1	0
7	C	243	0	0	1	0
7	D	269	0	0	1	0
All	All	16073	0	14770	49	0

The all-atom clashscore is defined as the number of clashes found per 1000 atoms (including hydrogen atoms). The all-atom clashscore for this structure is 2.

All (49) close contacts within the same asymmetric unit are listed below, sorted by their clash magnitude.

Atom-1	Atom-2	Interatomic distance (Å)	Clash overlap (Å)
1:D:370:ASP:OD2	1:D:375:ARG:NH1	2.25	0.69
1:B:80:ASP:OD2	7:B:601:HOH:O	2.17	0.59
1:C:217:ASP:OD1	1:C:255:ARG:NH1	2.35	0.57
1:C:59:LYS:NZ	7:C:603:HOH:O	2.37	0.57
1:D:148:LEU:HD21	1:D:413:VAL:HG21	1.89	0.54
1:B:68[A]:ASP:OD1	1:B:69:LYS:N	2.40	0.54
1:A:148:LEU:HD21	1:A:413:VAL:HG21	1.91	0.53
1:C:183:GLU:OE2	1:C:190:ARG:NH2	2.38	0.51
1:D:124:VAL:CG1	1:D:458:ILE:HD11	2.41	0.51
1:D:173:PHE:CD1	1:D:215:LEU:HD22	2.47	0.49
1:D:368:GLY:O	1:D:371:VAL:HG13	2.13	0.49
1:A:216:VAL:HG21	1:A:259:ILE:HG13	1.95	0.48
1:D:185:MET:HE1	1:D:437:LEU:HD11	1.94	0.48
1:B:388:HIS:HA	1:B:391:LYS:HD3	1.94	0.48
1:D:375:ARG:NH2	7:D:615:HOH:O	2.46	0.48
2:C:501:HEM:CMB	2:C:501:HEM:HBB2	2.44	0.47
1:A:141:VAL:HB	1:A:142:PRO:HD3	1.96	0.47

Continued on next page...

CONFIDENTIAL VALIDATION REPORT

Continued from previous page...

Atom-1	Atom-2	Interatomic distance (Å)	Clash overlap (Å)
1:D:68:ASP:OD1	1:D:69:LYS:N	2.48	0.46
1:C:141:VAL:HB	1:C:142:PRO:HD3	1.99	0.45
1:C:145[A]:MET:HE3	1:C:273:LEU:HD13	1.99	0.45
1:D:141:VAL:HB	1:D:142:PRO:HD3	1.99	0.45
1:A:83:GLY:HA2	5:A:510:GOL:H2	1.99	0.44
1:D:162:PHE:CE1	1:D:215:LEU:HD21	2.53	0.44
1:C:148:LEU:HD21	1:C:413:VAL:HG21	2.00	0.44
1:A:367:TRP:HB2	1:A:371:VAL:HG12	2.01	0.43
1:D:74:ALA:O	1:D:78:VAL:HG23	2.18	0.43
1:A:267[A]:VAL:HG13	1:A:268:THR:N	2.33	0.43
1:D:96:TRP:CZ2	1:D:398:ARG:HD2	2.53	0.43
1:C:213:ASN:OD1	1:C:259:ILE:HD11	2.18	0.43
1:B:278:TYR:HA	1:B:444:PHE:CZ	2.53	0.42
1:C:436:THR:O	1:C:437:LEU:HB2	2.19	0.42
1:A:250:ASP:OD2	7:A:601:HOH:O	2.21	0.42
1:A:76:LYS:O	1:A:79:ARG:HB2	2.20	0.42
1:B:298:LEU:HD22	1:B:303:PRO:HB3	2.00	0.42
1:C:253:ASN:O	1:C:257:GLN:HG2	2.20	0.42
1:A:216:VAL:HG21	1:A:259:ILE:CG1	2.50	0.42
1:A:39:ILE:HA	1:A:51:TYR:O	2.20	0.42
1:C:162:PHE:O	1:C:167[B]:ARG:NH1	2.53	0.41
1:C:366:ILE:HG21	1:C:389:ALA:HB1	2.01	0.41
2:B:501:HEM:HBC2	2:B:501:HEM:CMC	2.51	0.41
1:A:391:LYS:HE2	1:A:395:ASN:HB2	2.03	0.41
1:C:79:ARG:HG3	1:C:88:THR:HB	2.03	0.41
2:A:501:HEM:HBC2	2:A:501:HEM:CMC	2.51	0.40
2:C:501:HEM:HMB2	2:C:501:HEM:HBB2	2.03	0.40
2:D:501:HEM:CMB	2:D:501:HEM:HBB2	2.51	0.40
1:B:148:LEU:HD21	1:B:413:VAL:HG21	2.03	0.40
1:D:124:VAL:HG11	1:D:458:ILE:HD11	2.03	0.40
1:A:150:LEU:HD22	1:A:174:ILE:HD11	2.02	0.40
1:C:74:ALA:O	1:C:78:VAL:HG23	2.22	0.40

There are no symmetry-related clashes.

5.3 Torsion angles (i)

5.3.1 Protein backbone (i)

In the following table, the Percentiles column shows the percent Ramachandran outliers of the chain as a percentile score with respect to all X-ray entries followed by that with respect to entries

CONFIDENTIAL VALIDATION REPORT

of similar resolution.

The Analysed column shows the number of residues for which the backbone conformation was analysed, and the total number of residues.

Mol	Chain	Analysed	Favoured	Allowed	Outliers	Percentiles	
1	A	458/507 (90%)	444 (97%)	14 (3%)	0	100	100
1	B	455/507 (90%)	440 (97%)	15 (3%)	0	100	100
1	C	451/507 (89%)	439 (97%)	12 (3%)	0	100	100
1	D	446/507 (88%)	429 (96%)	16 (4%)	1 (0%)	49	48
All	All	1810/2028 (89%)	1752 (97%)	57 (3%)	1 (0%)	53	54

All (1) Ramachandran outliers are listed below:

Mol	Chain	Res	Type
1	D	196	PRO

5.3.2 Protein sidechains ⓘ

In the following table, the Percentiles column shows the percent sidechain outliers of the chain as a percentile score with respect to all X-ray entries followed by that with respect to entries of similar resolution.

The Analysed column shows the number of residues for which the sidechain conformation was analysed, and the total number of residues.

Mol	Chain	Analysed	Rotameric	Outliers	Percentiles	
1	A	401/439 (91%)	399 (100%)	2 (0%)	90	93
1	B	399/439 (91%)	398 (100%)	1 (0%)	93	95
1	C	395/439 (90%)	394 (100%)	1 (0%)	93	95
1	D	392/439 (89%)	391 (100%)	1 (0%)	93	95
All	All	1587/1756 (90%)	1582 (100%)	5 (0%)	93	95

All (5) residues with a non-rotameric sidechain are listed below:

Mol	Chain	Res	Type
1	A	79	ARG
1	A	148	LEU
1	B	380	GLU
1	C	148	LEU
1	D	148	LEU

CONFIDENTIAL VALIDATION REPORT

Some sidechains can be flipped to improve hydrogen bonding and reduce clashes. There are no such sidechains identified.

5.3.3 RNA (i)

There are no RNA molecules in this entry.

5.4 Non-standard residues in protein, DNA, RNA chains (i)

There are no non-standard protein/DNA/RNA residues in this entry.

5.5 Carbohydrates (i)

There are no carbohydrates in this entry.

5.6 Ligand geometry (i)

Of 35 ligands modelled in this entry, 20 are monoatomic - leaving 15 for Mogul analysis.

In the following table, the Counts columns list the number of bonds (or angles) for which Mogul statistics could be retrieved, the number of bonds (or angles) that are observed in the model and the number of bonds (or angles) that are defined in the Chemical Component Dictionary. The Link column lists molecule types, if any, to which the group is linked. The Z score for a bond length (or angle) is the number of standard deviations the observed value is removed from the expected value. A bond length (or angle) with $|Z| > 2$ is considered an outlier worth inspection. RMSZ is the root-mean-square of all Z scores of the bond lengths (or angles).

Mol	Type	Chain	Res	Link	Bond lengths			Bond angles		
					Counts	RMSZ	$\#\|Z\| > 2$	Counts	RMSZ	$\#\|Z\| > 2$
2	HEM	A	501	1,3	27,50,50	1.87	4 (14%)	17,82,82	1.49	3 (17%)
3	DTT	A	502	2	7,7,7	0.36	0	4,8,8	0.85	0
3	DTT	A	503	1	7,7,7	0.38	0	4,8,8	0.66	0
5	GOL	A	510	-	5,5,5	1.09	0	5,5,5	0.83	0
5	GOL	A	511	-	5,5,5	0.94	0	5,5,5	0.92	0
6	PEG	A	512	-	6,6,6	0.38	0	5,5,5	0.99	0
2	HEM	B	501	1,3	27,50,50	1.84	5 (18%)	17,82,82	1.80	4 (23%)
3	DTT	B	502	2	7,7,7	0.40	0	4,8,8	0.59	0
5	GOL	B	508	-	5,5,5	0.84	0	5,5,5	0.97	0
2	HEM	C	501	1,3	27,50,50	1.85	4 (14%)	17,82,82	1.56	2 (11%)
3	DTT	C	502	2	7,7,7	0.42	0	4,8,8	0.54	0
5	GOL	C	507	-	5,5,5	0.99	0	5,5,5	0.94	0
6	PEG	C	508	-	6,6,6	0.45	0	5,5,5	0.30	0

CONFIDENTIAL VALIDATION REPORT

Mol	Type	Chain	Res	Link	Bond lengths			Bond angles		
					Counts	RMSZ	#|Z| > 2	Counts	RMSZ	#|Z| > 2
2	HEM	D	501	1,3	27,50,50	1.79	4 (14%)	17,82,82	1.68	4 (23%)
3	DTT	D	502	2	7,7,7	0.51	0	4,8,8	0.80	0

In the following table, the Chirals column lists the number of chiral outliers, the number of chiral centers analysed, the number of these observed in the model and the number defined in the Chemical Component Dictionary. Similar counts are reported in the Torsion and Rings columns. '-' means no outliers of that kind were identified.

Mol	Type	Chain	Res	Link	Chirals	Torsions	Rings
2	HEM	A	501	1,3	-	0/6/54/54	0/0/8/8
3	DTT	A	502	2	-	0/8/8/8	0/0/0/0
3	DTT	A	503	1	-	0/8/8/8	0/0/0/0
5	GOL	A	510	-	-	0/4/4/4	0/0/0/0
5	GOL	A	511	-	-	0/4/4/4	0/0/0/0
6	PEG	A	512	-	-	0/4/4/4	0/0/0/0
2	HEM	B	501	1,3	-	0/6/54/54	0/0/8/8
3	DTT	B	502	2	-	0/8/8/8	0/0/0/0
5	GOL	B	508	-	-	0/4/4/4	0/0/0/0
2	HEM	C	501	1,3	-	0/6/54/54	0/0/8/8
3	DTT	C	502	2	-	0/8/8/8	0/0/0/0
5	GOL	C	507	-	-	0/4/4/4	0/0/0/0
6	PEG	C	508	-	-	0/4/4/4	0/0/0/0
2	HEM	D	501	1,3	-	0/6/54/54	0/0/8/8
3	DTT	D	502	2	-	0/8/8/8	0/0/0/0

All (17) bond length outliers are listed below:

Mol	Chain	Res	Type	Atoms	Z	Observed(Å)	Ideal(Å)
2	A	501	HEM	C3C-C2C	-4.55	1.34	1.40
2	B	501	HEM	C3B-C2B	-4.49	1.34	1.40
2	D	501	HEM	C3C-C2C	-4.37	1.34	1.40
2	C	501	HEM	C3C-C2C	-4.25	1.34	1.40
2	C	501	HEM	C3B-C2B	-4.22	1.34	1.40
2	B	501	HEM	C3C-C2C	-4.22	1.34	1.40
2	A	501	HEM	C3B-C2B	-4.20	1.34	1.40
2	D	501	HEM	C3B-C2B	-3.92	1.34	1.40
2	B	501	HEM	CAA-C2A	2.26	1.55	1.52
2	B	501	HEM	C3B-CAB	3.26	1.54	1.47
2	C	501	HEM	C3B-CAB	3.37	1.54	1.47
2	D	501	HEM	C3C-CAC	3.50	1.54	1.47
2	B	501	HEM	C3C-CAC	3.56	1.54	1.47
2	A	501	HEM	C3B-CAB	3.61	1.55	1.47

Continued on next page...

CONFIDENTIAL VALIDATION REPORT

Continued from previous page...

Mol	Chain	Res	Type	Atoms	Z	Observed(Å)	Ideal(Å)
2	D	501	HEM	C3B-CAB	3.71	1.55	1.47
2	A	501	HEM	C3C-CAC	3.77	1.55	1.47
2	C	501	HEM	C3C-CAC	3.86	1.55	1.47

All (13) bond angle outliers are listed below:

Mol	Chain	Res	Type	Atoms	Z	Observed(^{o})	Ideal(^{o})
2	B	501	HEM	CBD-CAD-C3D	-3.69	105.44	112.47
2	C	501	HEM	CBD-CAD-C3D	-3.02	106.71	112.47
2	D	501	HEM	CMD-C2D-C1D	-2.86	124.07	128.46
2	D	501	HEM	CBD-CAD-C3D	-2.70	107.32	112.47
2	B	501	HEM	CMA-C3A-C4A	-2.63	124.42	128.46
2	A	501	HEM	CBD-CAD-C3D	-2.62	107.47	112.47
2	B	501	HEM	CMD-C2D-C1D	-2.50	124.62	128.46
2	D	501	HEM	CMA-C3A-C4A	-2.45	124.69	128.46
2	C	501	HEM	CMD-C2D-C1D	-2.25	125.01	128.46
2	A	501	HEM	CMD-C2D-C1D	-2.19	125.10	128.46
2	A	501	HEM	C1D-C2D-C3D	2.02	108.40	107.00
2	D	501	HEM	CMB-C2B-C3B	2.63	129.67	124.88
2	B	501	HEM	C4A-C3A-C2A	2.87	108.99	107.00

There are no chirality outliers.

There are no torsion outliers.

There are no ring outliers.

5 monomers are involved in 6 short contacts:

Mol	Chain	Res	Type	Clashes	Symm-Clashes
2	A	501	HEM	1	0
5	A	510	GOL	1	0
2	B	501	HEM	1	0
2	C	501	HEM	2	0
2	D	501	HEM	1	0

5.7 Other polymers (i)

There are no such residues in this entry.

CONFIDENTIAL VALIDATION REPORT

5.8 Polymer linkage issues (i)

There are no chain breaks in this entry.

CONFIDENTIAL VALIDATION REPORT

6 Fit of model and data (i)

6.1 Protein, DNA and RNA chains (i)

In the following table, the column labelled '#RSRZ> 2' contains the number (and percentage) of RSRZ outliers, followed by percent RSRZ outliers for the chain as percentile scores relative to all X-ray entries and entries of similar resolution. The OWAB column contains the minimum, median, 95th percentile and maximum values of the occupancy-weighted average B-factor per residue. The column labelled 'Q< 0.9' lists the number of (and percentage) of residues with an average occupancy less than 0.9.

Mol	Chain	Analysed	<RSRZ>	#RSRZ>2			OWAB(Å^2)	Q<0.9
1	A	455/507 (89%)	-0.23	3 (0%)	87	90	10, 20, 44, 79	0
1	B	452/507 (89%)	-0.11	6 (1%)	77	81	11, 22, 45, 72	0
1	C	450/507 (88%)	-0.02	18 (4%)	38	46	13, 26, 59, 88	0
1	D	450/507 (88%)	-0.06	7 (1%)	72	77	11, 25, 59, 86	0
All	All	1807/2028 (89%)	-0.11	34 (1%)	66	73	10, 23, 53, 88	0

All (34) RSRZ outliers are listed below:

Mol	Chain	Res	Type	RSRZ
1	C	-3	ARG	5.0
1	C	-5	MET	4.8
1	C	185	MET	4.4
1	D	227	GLY	4.4
1	C	-2	GLY	4.0
1	C	206	GLN	3.4
1	C	189	GLN	3.4
1	B	246	GLY	3.3
1	C	227	GLY	3.3
1	D	437	LEU	3.3
1	B	245	THR	3.2
1	C	384	ALA	3.2
1	C	205	PHE	3.0
1	A	4	GLU	2.8
1	C	-1	SER	2.8
1	C	211	VAL	2.7
1	B	244	GLU	2.6
1	A	245	THR	2.6
1	D	200	GLU	2.6
1	B	455	LEU	2.6
1	C	457	GLY	2.3

Continued on next page...

CONFIDENTIAL VALIDATION REPORT

Continued from previous page...

Mol	Chain	Res	Type	RSRZ
1	C	182	ASP	2.3
1	B	243	PRO	2.3
1	C	191	ALA	2.3
1	D	188	GLN	2.3
1	C	209	ILE	2.3
1	A	456	GLY	2.2
1	D	207	GLU	2.2
1	C	186	ASN	2.2
1	B	247	GLU	2.1
1	D	243	PRO	2.1
1	C	77	PHE	2.1
1	D	206	GLN	2.0
1	C	110	GLN	2.0

6.2 Non-standard residues in protein, DNA, RNA chains (i)

There are no non-standard protein/DNA/RNA residues in this entry.

6.3 Carbohydrates (i)

There are no carbohydrates in this entry.

6.4 Ligands (i)

In the following table, the Atoms column lists the number of modelled atoms in the group and the number defined in the chemical component dictionary. The B-factors column lists the minimum, median, 95th percentile and maximum values of B factors of atoms in the group. The column labelled 'Q< 0.9' lists the number of atoms with occupancy less than 0.9.

Mol	Type	Chain	Res	Atoms	RSCC	RSR	B-factors(Å^2)	Q<0.9
6	PEG	A	512	7/7	0.78	0.29	72,73,76,77	0
3	DTT	B	502	8/8	0.78	0.17	50,54,59,63	0
5	GOL	B	508	6/6	0.80	0.21	52,54,54,57	0
6	PEG	C	508	7/7	0.81	0.18	65,69,72,73	0
3	DTT	D	502	8/8	0.83	0.19	54,59,64,68	0
5	GOL	A	510	6/6	0.84	0.21	36,46,49,50	0
5	GOL	A	511	6/6	0.86	0.24	64,66,67,68	0
3	DTT	C	502	8/8	0.86	0.21	48,55,63,68	0
5	GOL	C	507	6/6	0.87	0.20	56,58,61,62	0
3	DTT	A	502	8/8	0.87	0.19	50,55,60,64	0

Continued on next page...

CONFIDENTIAL VALIDATION REPORT

Continued from previous page...

Mol	Type	Chain	Res	Atoms	RSCC	RSR	B-factors(Å^2)	Q<0.9
4	CL	B	503	1/1	0.90	0.10	47,47,47,47	0
4	CL	B	504	1/1	0.90	0.17	57,57,57,57	0
3	DTT	A	503	8/8	0.92	0.16	34,44,49,54	0
4	CL	A	505	1/1	0.94	0.11	44,44,44,44	0
4	CL	C	503	1/1	0.94	0.12	54,54,54,54	0
4	CL	B	506	1/1	0.95	0.10	47,47,47,47	0
4	CL	C	504	1/1	0.95	0.12	52,52,52,52	0
4	CL	D	503	1/1	0.97	0.07	36,36,36,36	0
2	HEM	B	501	43/43	0.97	0.12	9,13,20,24	0
4	CL	A	508	1/1	0.97	0.08	28,28,28,28	0
4	CL	A	507	1/1	0.98	0.12	56,56,56,56	0
2	HEM	C	501	43/43	0.98	0.10	12,15,18,22	0
2	HEM	D	501	43/43	0.98	0.11	11,16,19,20	0
2	HEM	A	501	43/43	0.98	0.11	7,11,13,14	0
4	CL	A	504	1/1	0.98	0.13	52,52,52,52	0
4	CL	D	506	1/1	0.98	0.07	45,45,45,45	0
4	CL	D	507	1/1	0.99	0.06	33,33,33,33	0
4	CL	D	504	1/1	0.99	0.07	38,38,38,38	0
4	CL	B	507	1/1	0.99	0.07	27,27,27,27	0
4	CL	C	506	1/1	0.99	0.08	35,35,35,35	0
4	CL	D	505	1/1	0.99	0.06	40,40,40,40	0
4	CL	A	509	1/1	0.99	0.09	42,42,42,42	0
4	CL	C	505	1/1	0.99	0.05	44,44,44,44	0
4	CL	B	505	1/1	0.99	0.13	40,40,40,40	0
4	CL	A	506	1/1	1.00	0.07	29,29,29,29	0

6.5 Other polymers (i)

There are no such residues in this entry.

CONFIDENTIAL VALIDATION REPORT
