# Supplementary material for: Structural analysis of Cytochrome P450 BM3 mutant M11 in complex with dithiothreitol
Source: PLoS One. 2019 May 24;14(5):e0217292. doi: 10.1371/journal.pone.0217292 (PMC6534296; doi:10.1371/journal.pone.0217292)
Supplement: S3 File — Atomic coordinates, atom types and partial atomic charges of neutral and anionic DTT and the heme group. (PDF) [file pone.0217292.s012.pdf]

```
@<TRIPOS>MOLECULE
dtt_rr
18   17    1
SMALL
USER_CHARGES


@<TRIPOS>ATOM
      1 S1          0.8537    1.0225   -6.1877 S.3       1 DTU         -0.3672
      2 C1          1.4512    0.6370   -4.5143 C.3       1 DTU         -0.0937
      3 C2          2.2583    1.8117   -3.9118 C.3       1 DTU          0.2243
      4 O2          3.5313    1.8883   -4.5363 O.3       1 DTU         -0.6495
      5 C3          2.4053    1.7183   -2.3672 C.3       1 DTU          0.2243
      6 O3          1.3176    2.3591   -1.7171 O.3       1 DTU         -0.6495
      7 C4          2.5326    0.2623   -1.8590 C.3       1 DTU         -0.0937
      8 S4          4.0962   -0.4990   -2.3892 S.3       1 DTU         -0.3672
      9 HS1         1.6956    2.0358   -6.4096 H         1 DTU          0.1813
     10 H11         2.0481   -0.2751   -4.5508 H         1 DTU          0.0998
     11 H12         0.5795    0.4180   -3.8967 H         1 DTU          0.0998
     12 H2          1.7296    2.7384   -4.1418 H         1 DTU          0.0757
     13 HO2         3.4790    1.4586   -5.3931 H         1 DTU          0.4293
     14 H3          3.3123    2.2557   -2.0847 H         1 DTU          0.0757
     15 HO3         1.1787    3.2168   -2.1250 H         1 DTU          0.4293
     16 H41         1.7197   -0.3498   -2.2509 H         1 DTU          0.0998
     17 H42         2.4649    0.2160   -0.7713 H         1 DTU          0.0998
     18 HS4         3.8279   -1.7194   -1.9159 H         1 DTU          0.1813
@<TRIPOS>BOND
     1    1    2 1
     2    1    9 1
     3    2    3 1
     4    2   10 1
     5    2   11 1
     6    3    4 1
```

```
     7    3     5 1
     8    3    12 1
     9    4    13 1
    10    5     6 1
    11    5     7 1
    12    5    14 1
    13    6    15 1
    14    7     8 1
    15    7    16 1
    16    7    17 1
    17    8    18 1
@<TRIPOS>SUBSTRUCTURE
     1 DTU         1 GROUP             0       ****    0 ROOT


----------

@<TRIPOS>MOLECULE
dtt_rr_neg
17   16    1
SMALL
USER_CHARGES


@<TRIPOS>ATOM
      1 S1          5.0895   -2.1450   -2.3350 S.3       1 DTU        -0.4082
      2 C1          4.6341   -0.4629   -2.8535 C.3       1 DTU        -0.0784
      3 C2          3.1213   -0.3494   -3.1581 C.3       1 DTU         0.2138
      4 O2          2.7149   -1.4355   -3.9777 O.3       1 DTU        -0.6469
      5 C3          2.7328    1.0084   -3.8073 C.3       1 DTU         0.1504
      6 O3          1.3415    1.0540   -4.0876 O.3       1 DTU        -0.6571
      7 C4          3.1313    2.2280   -2.9424 C.3       1 DTU        -0.0089
      8 S4          2.3062    2.2000   -1.3226 S.3       1 DTU        -0.9601
      9 HS1         4.7971   -1.9795   -1.0419 H         1 DTU         0.1633
```

```
     10 H11          5.2339   -0.1829   -3.7203 H         1 DTU          0.1030
     11 H12          4.9116    0.2160   -2.0464 H         1 DTU          0.1030
     12 H2           2.5789   -0.4362   -2.2148 H         1 DTU          0.0443
     13 HO2          1.9992   -1.9004   -3.5383 H         1 DTU          0.4089
     14 H3           3.2571    1.0847   -4.7616 H         1 DTU          0.0668
     15 HO3          0.8636    0.7265   -3.3223 H         1 DTU          0.4152
     16 H41          2.8475    3.1560   -3.4397 H         1 DTU          0.0455
     17 H42          4.2108    2.2695   -2.7914 H         1 DTU          0.0455
@<TRIPOS>BOND
     1    1    2 1
     2    1    9 1
     3    2    3 1
     4    2   10 1
     5    2   11 1
     6    3    4 1
     7    3    5 1
     8    3   12 1
     9    4   13 1
    10    5    6 1
    11    5    7 1
    12    5   14 1
    13    6   15 1
    14    7    8 1
    15    7   16 1
    16    7   17 1
@<TRIPOS>SUBSTRUCTURE
     1 DTU          1 GROUP             0       ****    0 ROOT


----------

@<TRIPOS>MOLECULE
heme
73   80    1
```

```
SMALL

USER_CHARGES


@<TRIPOS>ATOM

      1 CAA          8.3495   -0.6205   -1.1049 C.3       1 HEM        -0.0738

      2 CAB         -0.0301    2.3016    3.2735 C.2       1 HEM        -0.1859

      3 CAC          2.5605    8.4845   -0.9686 C.2       1 HEM        -0.1859

      4 CAD          8.7861    4.2779   -3.4989 C.3       1 HEM        -0.0738

      5 NA           5.4716    1.5314   -0.1380 N.2       1 HEM        -0.4980

      6 CBA          8.1108   -1.3613   -2.4463 C.3       1 HEM        -0.2200

      7 CBB         -0.4160    1.7206    4.4237 C.2       1 HEM        -0.2300

      8 CBC          2.0261    9.5616   -0.3653 C.2       1 HEM        -0.2300

      9 CBD         10.0535    4.6648   -2.6932 C.3       1 HEM        -0.2200

     10 NB           2.9783    2.3566    1.1765 N.pl3     1 HEM        -0.4980

     11 CGA          9.3411   -2.0989   -2.9858 C.2       1 HEM         0.7000

     12 CGD         11.3628    4.5359   -3.4795 C.2       1 HEM         0.7000

     13 ND           5.7350    4.1529   -1.3790 N.pl3     1 HEM        -0.4980

     14 CHA          7.2486    2.2751   -1.6190 C.ar      1 HEM        -0.1500

     15 CHB          4.0666    0.2000    1.3423 C.ar      1 HEM        -0.1500

     16 CHC          1.4865    4.2438    1.4260 C.ar      1 HEM        -0.1500

     17 CHD          4.6626    6.3227   -1.5341 C.ar      1 HEM        -0.1500

     18 CMA          6.2026   -1.9829    0.7997 C.3       1 HEM        -0.1338

     19 CMB          1.6319   -0.4652    3.1680 C.3       1 HEM        -0.1338

     20 CMC          0.3660    7.0953    0.9383 C.3       1 HEM        -0.1338

     21 CMD          7.0887    6.9648   -3.3772 C.3       1 HEM        -0.1338

     22 C1A          6.6222    1.3240   -0.8076 C.ar      1 HEM         0.1003

     23 C1B          3.0433    1.1008    1.6492 C.ar      1 HEM         0.1003

     24 C1C          2.1102    5.1944    0.6115 C.ar      1 HEM         0.1003

     25 C1D          5.6762    5.4128   -1.8412 C.ar      1 HEM         0.1003

     26 O1A         10.4157   -2.0812   -2.3524 O.co2     1 HEM        -0.8000

     27 O1D         12.4178    4.8242   -2.8803 O.co2     1 HEM        -0.8000

     28 C2A          7.0800    0.0373   -0.5680 C.ar      1 HEM        -0.0280
```

29	C2B	1.9536	0.8520	2.4696	C.ar	1	HEM	-0.0280
30	C2C	1.6494	6.4827	0.3853	C.ar	1	HEM	-0.0280
31	C2D	6.7657	5.6611	-2.6589	C.ar	1	HEM	-0.0280
32	O2A	9.2114	-2.7088	-4.0659	O.co2	1	HEM	-0.8000
33	O2D	11.3499	4.1619	-4.6696	O.co2	1	HEM	-0.8000
34	C3A	6.1543	-0.5573	0.2642	C.ar	1	HEM	-0.0280
35	C3B	1.2118	2.0206	2.5115	C.ar	1	HEM	0.0891
36	C3C	2.5654	7.0926	-0.4535	C.ar	1	HEM	0.0891
37	C3D	7.5010	4.4940	-2.7034	C.ar	1	HEM	-0.0280
38	C4A	5.1809	0.3939	0.5210	C.ar	1	HEM	0.1003
39	C4B	1.8743	2.9261	1.6910	C.ar	1	HEM	0.1003
40	C4C	3.5375	6.1362	-0.7221	C.ar	1	HEM	0.1003
41	C4D	6.8430	3.5850	-1.8889	C.ar	1	HEM	0.1003
42	NC	3.2509	4.9899	-0.0730	N.2	1	HEM	-0.4980
43	FE	4.3591	3.2943	-0.1192	Fe	1	HEM	1.1904
44	HAA1	9.1282	0.1352	-1.2180	H	1	HEM	0.0600
45	HAA2	8.7288	-1.3103	-0.3491	H	1	HEM	0.0600
46	HAB	-0.6942	3.0360	2.8391	H	1	HEM	0.1150
47	HAC	3.0177	8.6308	-1.9374	H	1	HEM	0.1150
48	HAD1	8.8406	3.2371	-3.8215	H	1	HEM	0.0600
49	HAD2	8.7252	4.8606	-4.4197	H	1	HEM	0.0600
50	HBA1	7.7798	-0.6514	-3.2050	H	1	HEM	0.0600
51	HBA2	7.3110	-2.0927	-2.3257	H	1	HEM	0.0600
52	HBB1	-1.3541	1.9876	4.8906	H	1	HEM	0.1150
53	HBB2	0.1864	0.9788	4.9276	H	1	HEM	0.1150
54	HBC1	2.0611	10.5342	-0.8366	H	1	HEM	0.1150
55	HBC2	1.5517	9.5047	0.6038	H	1	HEM	0.1150
56	HBD1	10.1285	4.0403	-1.8024	H	1	HEM	0.0600
57	HBD2	9.9718	5.6970	-2.3512	H	1	HEM	0.0600
58	HHA	8.1633	1.9590	-2.0959	H	1	HEM	0.1134
59	HHB	3.9943	-0.7722	1.8042	H	1	HEM	0.1134
60	HHC	0.5869	4.5717	1.9214	H	1	HEM	0.1134

```
     61 HHD          4.7666    7.2971   -1.9854 H          1 HEM          0.1134
     62 HMA1         5.2207   -2.4522    0.7367 H          1 HEM          0.0600
     63 HMA2         6.8947   -2.6057    0.2336 H          1 HEM          0.0600
     64 HMA3         6.5178   -1.9828    1.8432 H          1 HEM          0.0600
     65 HMB1         0.5549   -0.6113    3.2484 H          1 HEM          0.0600
     66 HMB2         2.0202   -1.3206    2.6165 H          1 HEM          0.0600
     67 HMB3         2.0613   -0.4786    4.1697 H          1 HEM          0.0600
     68 HMC1        -0.0468    7.8297    0.2472 H          1 HEM          0.0600
     69 HMC2        -0.4083    6.3437    1.0878 H          1 HEM          0.0600
     70 HMC3         0.5597    7.5850    1.8926 H          1 HEM          0.0600
     71 HMD1         8.1255    6.9974   -3.7108 H          1 HEM          0.0600
     72 HMD2         6.4450    7.0841   -4.2488 H          1 HEM          0.0600
     73 HMD3         6.9344    7.8179   -2.7163 H          1 HEM          0.0600
@<TRIPOS>BOND
     1     1     6 1
     2     1    28 1
     3     1    44 1
     4     1    45 1
     5     2     7 2
     6     2    35 1
     7     2    46 1
     8     3     8 2
     9     3    36 1
    10     3    47 1
    11     4     9 1
    12     4    37 1
    13     4    48 1
    14     4    49 1
    15     5    22 ar
    16     5    38 ar
    17     5    43 nc
    18     6    11 1
```

19	6	50	1
20	6	51	1
21	7	52	1
22	7	53	1
23	8	54	1
24	8	55	1
25	9	12	1
26	9	56	1
27	9	57	1
28	10	23	ar
29	10	39	ar
30	10	43	nc
31	11	26	2
32	11	32	1
33	12	27	2
34	12	33	1
35	13	25	ar
36	13	41	ar
37	13	43	nc
38	14	22	ar
39	14	41	ar
40	14	58	1
41	15	23	ar
42	15	38	ar
43	15	59	1
44	16	24	ar
45	16	39	ar
46	16	60	1
47	17	25	ar
48	17	40	ar
49	17	61	1
50	18	34	1

```
    51   18   62 1
    52   18   63 1
    53   18   64 1
    54   19   29 1
    55   19   65 1
    56   19   66 1
    57   19   67 1
    58   20   30 1
    59   20   68 1
    60   20   69 1
    61   20   70 1
    62   21   31 1
    63   21   71 1
    64   21   72 1
    65   21   73 1
    66   22   28 ar
    67   23   29 ar
    68   24   30 ar
    69   24   42 ar
    70   25   31 ar
    71   28   34 ar
    72   29   35 ar
    73   30   36 ar
    74   31   37 ar
    75   34   38 ar
    76   35   39 ar
    77   36   40 ar
    78   37   41 ar
    79   40   42 ar
    80   42   43 nc
@<TRIPOS>SUBSTRUCTURE
     1 HEM         1 GROUP             0 E     ****    0 ROOT
```
